# Identification of influential proteins in the classical retinoic acid signaling pathway

**DOI:** 10.1186/s12976-018-0088-7

**Published:** 2018-10-16

**Authors:** Hamed Ghaffari, Linda R. Petzold

**Affiliations:** 1 Department of Mechanical Engineering, University of California Santa Barbara, Santa Barbara, CA 93106 USA; 2 Department of Computer Science, University of California Santa Barbara, Santa Barbara, CA 93106 USA

**Keywords:** Retinoic acid, Cellular retinoic acid binding protein, Retinoic acid signaling pathway, Retinoic acid receptor, Mathematical model, Global sensitivity analysis, Sobol’s method, Cytochrome P450

## Abstract

**Background:**

In the classical pathway of retinoic acid (RA) mediated gene transcription, RA binds to a nuclear hormone receptor dimer composed of retinoic acid receptor (RAR) and retinoid X receptor (RXR), to induce the expression of its downstream target genes. In addition to nuclear receptors, there are other intracellular RA binding proteins such as cellular retinoic acid binding proteins (CRABP1 and CRABP2) and cytochrome P450 (CYP) enzymes, whose contributions to the RA signaling pathway have not been fully understood. The objective of this study was to compare the significance of various RA binding receptors, i.e. CRABP1, CRABP2, CYP and RAR in the RA signaling pathway. In this regard, we developed a mathematical model of the RA pathway, which is one of the few models, if not the only one, that includes all main intracellular RA binding receptors. We then performed a global sensitivity analysis (GSA) to investigate the contribution of the RA receptors to RA-induced mRNA production, when the cells were treated with a wide range of RA levels, from physiological to pharmacological concentrations.

**Results:**

Our results show that CRABP2 and RAR are the most and the least important proteins, respectively, in controlling the model performance at physiological concentrations of RA (1–10 nM). However, at higher concentrations of RA, CYP and RAR are the most sensitive parameters of the system. Furthermore, we found that depending on the concentrations of all RA binding proteins, the rate of metabolism of RA can either change or remain constant following RA therapy. The cellular levels of CRABP1 are more important than that of CRABP2 in controlling RA metabolite formation at pharmacological conditions (RA = 0.1–1 μM). Finally, our results indicate a significant negative correlation between total mRNA production and total RA metabolite formation at pharmacological levels of RA.

**Conclusions:**

Our simulations indicate that the significance of the RA binding proteins in the RA pathway of gene expression strongly depends on intracellular concentration of RA. This study not only can explain why various cell types respond to RA therapy differently, but also can potentially help develop pharmacological methods to increase the efficacy of the drug.

**Electronic supplementary material:**

The online version of this article (10.1186/s12976-018-0088-7) contains supplementary material, which is available to authorized users.

## Background

Retinoic acid (RA), a biologically active form of vitamin A, plays essential roles in the growth and development of various cell types. RA has also been widely used as an anticancer drug due to its ability to inhibit cancer cell growth and induce cell differentiation. It is believed that RA mainly exerts its effects by regulating gene expression. The classical pathway of RA-induced gene transcription involves binding of RA to retinoic acid receptor (RAR), a member of the nuclear hormone family. The liganded RAR binds as a heterodimer (RA:RAR:RXR) to DNA and regulates gene expression. RAR:RXR heterodimer is the main transcription factor in the classical RA signaling pathway. The formation rate of RA:RAR:RXR complex, is highly affected by other intracellular RA binding receptors such as cellular retinoic acid binding proteins (CRABPs) and cytochrome P450 (CYP) enzymes. CRABPs are high affinity cytosolic receptors for RA that can potentially limit the access of RA to the RARs; CRABP1 and CRABP2 are the main members of the CRABP family. It has been reported that CRABP1 is responsible for sequestering RA in the cytosol, and thus controlling the level of free intracellular RA available for binding to RARs [1]. CRABP1 can also facilitate RA degradation by directing RA molecules to RA-degrading enzymes, cytochrome P450 (CYP) [2]. However, other in vitro studies have indicated that CRABP1 is dispensable in the RA signaling pathway [3, 4]. CRABP2, whose expression pattern is different from CRABP1 [5], delivers RA to both nuclear hormone receptors and CYP enzymes [6, 7]. CRABPs are bound to CYPs prior to adding RA to the cell [6, 8].

CYP enzymes are the main components of the pathway by which RA is cleared from the body. It is believed that liver cells which express high levels of CYP enzymes mainly mediate the synthesis and the clearance of RA [9, 10]. However, CYPs are found at various expression levels across different tissues and cell types [10]. Even though CRABP1, CRABP2, RAR and CYP are the main RA binding proteins, little is known about their expression levels across different human cell types. It is important to note that the cellular level of a protein can also vary considerably from cell to cell within a population of cells of the same type. Furthermore, to the best of our knowledge, the extent of contribution of the RA binding receptors to RA-induced gene transcription has yet to be elucidated. Understanding the roles and significance of RA binding receptors in the RA signaling pathway is important since it can help in the development of pharmacological approaches to limit or induce the activity of RA binding receptors, with the aim of increasing drug efficacy. Few previous in vitro studies have investigated the impacts of overexpression of CRABP1 and CRABP2 on RA-induced gene expression [3, 11]. However, their results were cell type-dependent, since different cell types have different expression levels of RA binding receptors. Furthermore, it is not clear whether the significance of the RA binding receptors in the RA pathway of gene expression depends on the RA concentration. In this study, we developed a new mathematical model to investigate the importance of various RA binding receptors in the RA signaling pathway in broad regions of RA concentrations. In this regard, we used a variance-based global sensitivity analysis (GSA) technique called Sobol’s method [12], which assesses the impacts of the model’s unknown parameters and the interactions between them on the model output. Total mRNA production and total RA metabolite formation within 24 h after RA treatment were selected as the model outputs, while the unknown parameters included kinetic rate constants and total concentrations of the RA binding receptors. Our results showed that all RA binding receptors could potentially influence mRNA production and RA metabolite formation by the RA pathway. However, the impact of a particular RA binding receptor on the model response largely depends on the concentrations of all RA binding receptors.

The main advantage of the current study over previous in vitro studies is that our results were obtained using wide ranges of RA receptor concentrations for any given RA concentration, thus our results are applicable to most cell types or to a population of cells of the same type. Furthermore, our study is able to reveal the synergistic effects of a combination of parameters across a broad range of parameter values. In contrast, the obtained results from previous experimental studies [3, 11] reveal the sensitivity of the system with respect to one parameter when the rest of the parameters remain unchanged.

## Methods

### Model development

We formulated a well-mixed ODE model of the RA signaling pathway. The model consisted of 17 species, which included proteins, mRNAs, protein-protein complexes and RA (Table 1).

**Table 1:** List of the model parameters

Parameter	Description
RA	Retinoic acid
CRABP1	Cellular retinoic acid binding protein 1
RA:CRABP1	Holo-cellular retinoic acid binding protein 1
CRABP2	Cellular retinoic acid binding protein 2
RA:CRABP2	Holo-cellular retinoic acid binding protein 2
CYP	CYP enzyme
RA:CYP	Liganded CYP
RAR	Retinoic acid receptor
RA:RAR	Activated retinoic acid receptor
RA:CRABP1:CYP	Activated CRABP1-CYP complex
RA:CRABP2:CYP	Activated CRABP2-CYP complex
RA:CRABP2:RAR	Activated CRABP2-RAR complex
CRABP1:CYP	CRABP1-CYP complex
CRABP2:CYP	CRABP2-CYP complex
*CRABP*2_*mRNA*_	Cellular retinoic acid binding protein 2 mRNA
*CYP*_*mRNA*_	CYP enzyme mRNA
*RAR*_*mRNA*_	Retinoic acid receptor mRNA

**Fig. 1:**
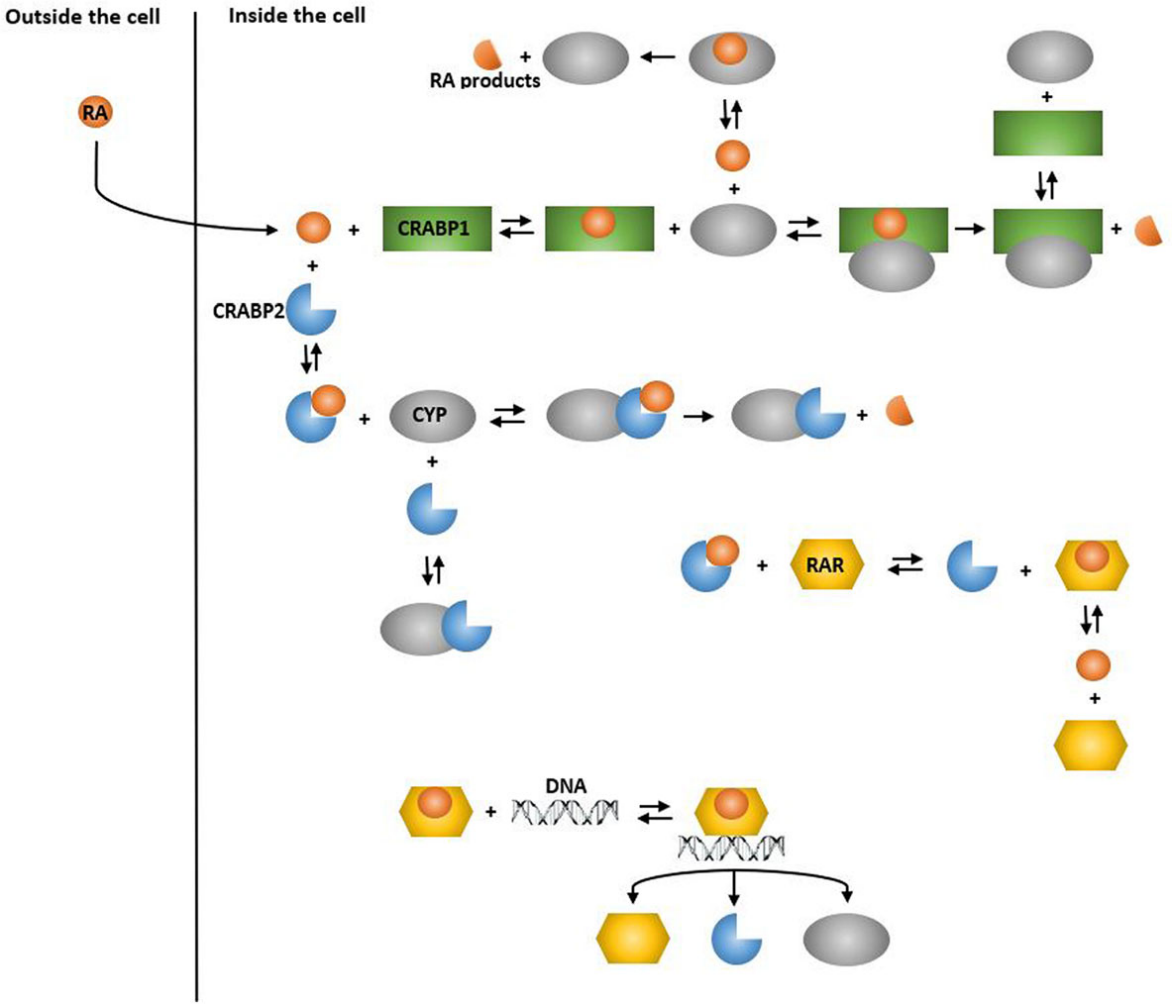
Simplified schematic of RA signaling pathway. CRABP1 is shown in green, while CRABP2 is shown in blue. Red circles, gray ellipsoids and yellow hexagons represent RA molecules, CYP enzymes and RAR molecules, respectively

The model included gene transcription, protein translation, and degradation of mRNA and protein. The model involved the mechanisms by which RA is degraded. The core set of reactions describing the RA metabolism process were taken directly from [6]. We simulated RA-induced gene transcription through the interactions between liganded transcription factor and DNA (Additional file 1). The model also describes how RA binding receptors interact with each other in the absence or presence of RA (Fig. 1). In the absence of RA, CRABPs complex with CYP enzymes, while RARs are not bound to CRABPs or CYPs [6, 8]. Once RA diffuses into the cell, it binds to different RA binding receptors with various binding affinities. CRABP1, which has the highest binding affinity for RA compared to the other RA receptors, regulates the metabolic fate of RA by directing RA molecules to CYP enzymes. In theory, CRABP1 can also transport RA to RAR. This process involves dissociation of RA from CRABP1, followed by association of RA with RAR. CRABP2 is the second high-affinity receptor for RA [5] and can deliver RA to RAR and CYP. RA is transported from CRABP2 to RAR by a mechanism that involves direct interactions between CRABP2 and RAR [5].

RA is transferred to CYP enzymes either freely or bound to CRABPs. RA-induced gene transcription depends on the rate of transfer of RA to RAR. Free RA molecules can interact with RARs directly. CRABP1 and CRABP2 can also deliver RA to RAR by different mechanisms. Liganded transcription factors can enhance the transcriptional activation of *CYP*, *RAR* and *CRABP2* genes after binding to DNA at a retinoic acid response element (RARE). We also assumed that RA was degraded only by CYP, while RA binding receptors, i.e. RAR, CRABP1, CRABP2, CYP were degraded by first-order reactions.

The full list of reactions in our model is presented in Table 2.

**Table 2:** List of reactions in the RA signaling pathway

Number	Reaction
1	RA + CRABP1 ⇔ RA : CRABP1
2	RA + CRABP2 ⇔ RA : CRABP2
3	RA + CYP ⇔ RA : CYP
4	RA : CYP ⇒ CYP + (RA metabolites)
5	RA + RAR ⇔ RA : RAR
6	*RAR*_mRNA_ ⇒ RAR
7	*CRABP*2_mRNA_ ⇒ CRABP2
8	*CYP*_mRNA_ ⇒ CYP
9	RA : CRABP1 + CYP ⇔ RA : CRABP1 : CYP
10	RA : CRABP1 : CYP ⇒ CRABP1 : CYP + (RA metabolites)
11	RA : CRABP2 + CYP ⇔ RA : CRABP2 : CYP
12	RA : CRABP2 : CYP ⇒ CRABP2 : CYP + (RA metabolites)
13	RA : CRABP2 + RAR ⇔ RA : CRABP2 : RAR
14	RA : CRABP2 : RAR ⇒ RA : RAR + CRABP2
15	CRABP1 + CYP ⇔ CRABP1 : CYP
16	CRABP2 + CYP ⇔ CRABP2 : CYP
17	CRABP1 ⇒ ∅
18	CRABP2 ⇒ ∅
19	CYP ⇒ ∅
20	RAR ⇒ ∅
21	RA : CRABP1 ⇒ RA + ∅
22	RA : CRABP2 ⇒ RA + ∅
23	RA : RAR ⇒ RA + ∅
24	*RAR*_mRNA_ ⇒ ∅
25	*CRABP*2_mRNA_ ⇒ ∅
26	*CYP*_mRNA_ ⇒ ∅
27	CRABP1 : CYP ⇒ CYP + ∅
28	CRABP1 : CYP ⇒ CRABP1 + ∅
29	CRABP2 : CYP ⇒ CYP + ∅
30	CRABP2 : CYP ⇒ CRABP2 + ∅

RA-induced expression of *RAR, CRBAP2* and *CYP* genes are modeled using Eq. 4

Analysis of the model behavior required the initial concentrations of the species and the kinetic parameters. Our model had 44 parameters, which included total concentrations of the RA binding receptors, the kinetic rate constants for binding/unbinding reactions, transcription and translation rate constants and mRNA and protein degradation rates. We assumed that total concentrations of CRABP1, CRABP2, CYP and RAR were unknown, which implies that these proteins are expressed at various levels across different tissues and across a population of cells of the same type. In the absence of RA, total concentrations of RA receptors were given by

1 $$ \left[\mathrm{CRABP}{1}_t\right]=\kern0.5em \left[\mathrm{CRABP}{1}_f\right]+\kern0.5em \left[\mathrm{CRABP}1:\mathrm{CYP}\right] $$

2 $$ \left[\mathrm{CRABP}{2}_t\right]=\kern0.5em \left[\mathrm{CRABP}{2}_f\right]+\kern0.5em \left[\mathrm{CRABP}2:\mathrm{CYP}\right] $$

3 $$ \left[{\mathrm{CYP}}_t\right]=\kern0.5em \left[{\mathrm{CYP}}_f\right]+\kern0.5em \left[\mathrm{CRABP}1:\mathrm{CYP}\right]+\kern0.5em \left[\mathrm{CRABP}2:\mathrm{CYP}\right] $$

where [] indicates molar concentration, while subscripts *t* and *f* stand for total and free receptors. RAR does not have any interaction with the remainder of the RA binding receptors, i.e. CRABP1, CRABP2 and CYP, before RA treatment. However, RAR can homodimerize, and heterodimerize with other proteins such as RXR in the absence of RA. In this study, we assumed that RA molecules can bind to free RARs, and to RARs bound to other proteins, with the same binding affinity. Thus, all RARs are receptive to RA binding.

We used in vitro values for 30 model parameters (see Additional file 1), while the remaining 14 parameters were unknown for which we considered some physiological bounds (Table 3). We also assumed that for a given gene the values of transcription rate constants, translation rate constants, forward and reverse rate constants of the binding reactions and the elimination rates of proteins and mRNAs can vary within the in vitro values by a factor of two. This is because not only can these parameters vary across cell type and across cells of the same type, but also all in vitro parameters are subject to error.

**Table 3:** List of the independent model parameters

Parameter	Description	Range	Reference
CRABP1	Total concentration of CRABP1	1 nM – 10 μM	Unknown. A large range is used.
CRABP2	Total concentration of CRABP2	1 nM – 10 μM	Unknown. A large range is used.
CYP	Total concentration of CYP	1 nM – 10 μM	Unknown. A large range is used.
RAR	Total concentration of RAR	1 nM – 1 μM	Unknown. A large range is used.
*k*_*d*3_	Equilibrium dissociation constant of reaction #3	1 nM −64 nM	[6]
*k*_*on*13_	Forward rate of reaction #13	3.6 × 10^9^ − 3.6 × 10^10^	[37]
*k*_*d*13_	Equilibrium dissociation constant of reaction # 13	0.1 nM–10 nM	[38]
*k*_*on*14_	Forward rate of reaction #14	50–200 1/h	[5]
*k*_*on*15_	Forward rate of reaction #15	3.6 × 10^9^ − 3.6 × 10^10^	[37]
*k*_*on*16_	Forward rate of reaction #16	3.6 × 10^9^ − 3.6 × 10^10^	[37]
*f*_*RAR*_	Transcription factor fraction for *RAR* gene	0–1	By definition
*f*_*CRABP*2_	Transcription factor fraction of *CRABP2* gene	0–1	By definition
*f*_*CYP*_	Transcription factor fraction for *CYP* gene	0–1	By definition
*f*_*GOI*_	Transcription factor fraction for the GOI	0–1	By definition

Reactions are shown in Table 2

Transcription factor fractions are defined in Section “Gene expression through RA pathway”

We used large ranges for unknown initial concentrations of CRABP1, CRABP2, CYP and RAR [13]. This is because the cellular levels of these proteins can vary significantly across cell types, or in a particular cell type as a consequence of cancer and cancer therapy. We then performed a global sensitivity analysis to identify the influential unknown parameters in the RA signaling pathway.

### Global sensitivity analysis of the model

Global sensitivity analysis (GSA) is a numerical technique designed to analyze the impacts of uncertain parameters on a model’s output. In contrast to local sensitivity analysis which analyzes the changes of model output by making small changes to each parameter while keeping the remaining parameters unchanged [14], GSA considers variations of all parameters over their entire range. Thus, GSA is useful for understanding the contribution of various model parameters to the variations in model output. In this study, we used a MATLAB toolbox for global sensitivity analysis, called SAFE [15]. We used a variance-based sensitivity analysis approach called Sobol’s method, which can quantitatively rank the relative importance of the model’s parameters [12]. Sobol’s method evaluates the first- and total-order sensitivity indices for each parameter. The first-order index (*S*_*i*_) represents the individual effects of each input on the variance of the output, while the total-effect index (*S*_*Ti*_) accounts for the total contribution of the input that includes its first-order effect plus all higher-order effects. The higher-order effects for a given input are due to interactions of the input with other model inputs. The total-effect sensitivity indices are useful in identifying the noninfluential parameters which can be fixed anywhere over their range of variability without influencing the output significantly [12]. If *S*_*Ti*_ ≤0.01 and the total-effect index of *x*_*i*_ is much smaller than that of the rest of parameters, then *x*_*i*_ can be fixed at any value within its range [16–18].

## Results

### Gene expression through RA pathway

We investigated the importance of various RA binding receptors in the RA signaling pathway after treating the model with various concentrations of RA. In this regard, we calculated the total mRNA production by a gene of interest (GOI) within 24 h after RA therapy. The rate of production of a mRNA of interest by the classical RA signaling pathway is modeled by (see Additional file 1 for details)

4 $$ \frac{\mathrm{d}\left[\mathrm{mRNA}\right]}{\mathrm{d}\mathrm{t}}=\kern0.5em {I}_{\max (GOI)}\kern0.5em \left(\frac{f_{GOI}\left[\mathrm{RA}:\mathrm{RAR}\right]}{f_{GOI}\left[\mathrm{RA}:\mathrm{RAR}\right]+{k}_{d\left( TF: DNA\right)}}\right) $$

where *I*_max*(GOI)*_ and *k*_*d*(*TF:DNA*)_ are the maximal transcription rate by an activated transcription factor (TF_*α*_) which initiates the transcription of the mRNA’s gene, and the equilibrium dissociation constant of binding of the transcription factor to DNA, respectively. *f*_*GOI*_ is the transcription factor fraction of the GOI, defined as the ratio of the concentration of total transcription factor (TF_t_) to the concentration of total RAR (RAR_t_),

5 $$ {f}_{GOI}=\kern0.5em \frac{\left[{\mathrm{TF}}_t\right]}{\left[{\mathrm{RAR}}_t\right]} $$

and is a number between 0 and 1. TF_t_ represents those heterodimerized RAR isotypes which can activate the transcription of the GOI after binding to RA. Some RA target genes can be expressed by various RAR:RXR heterodimers, while others are expressed by a particular heterodimer. Thus, the concentration of total transcription factor (TF_t_) is less than or equal to total concentration of RAR (RAR_t_). In general, the value of *f*_*GOI*_ depends on gene- and cell-type. For a given cell-type, *f*_*GOI*_ varies for different genes since the value of TF_t_ depends on gene-type.

**Fig. 2:**
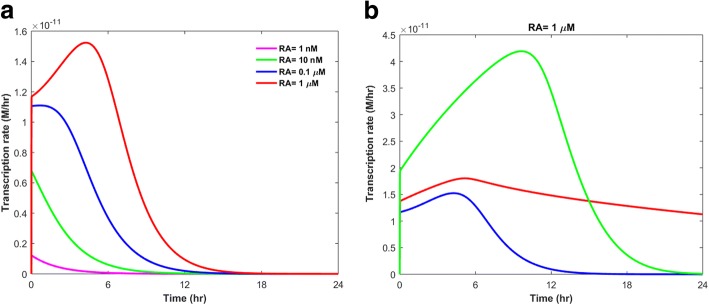
Changes in the transcription rate of the GOI (**a**) after adding various concentrations of RA to a model with a set of random parameters: [CRABP1_*t*_]=2.6 nM, [CRABP2_*t*_]=3.2 μM, [CYP_*t*_]=0.1μM, [RAR_*t*_]=3 nM. **b** after adding 1 μM of RA to various models with different sets of parameters; Green: [CRABP1_*t*_]=1 nM, [CRABP2_*t*_]=7.7 μM, [CYP_*t*_]=3.5 nM, [RAR_*t*_]=9 nM. Red: [CRABP1_*t*_]=10 nM, [CRABP2_*t*_]=1.9 μM, [CYP_*t*_]=15 nM, [RAR_*t*_]=0.17 μM. Blue: [CRABP1_*t*_]=2.6 nM, [CRABP2_*t*_]=3.2 μM, [CYP_*t*_]=0.1 μM, [RAR_*t*_]=3 nM

Figure 2a shows the variations in the transcription rate of the GOI within 24 h after adding various concentrations of RA to a model with a randomly sampled set of parameters.

The RA-induced transcription rate strongly depends on RA concentration and model parameters, i.e. initial concentrations of the RA receptors and kinetic rate constants (Fig. 2). The transcription rate peak time, duration of transcription, and transcription rate peak level can change or remain unchanged after modifying RA concentration or model parameters. In order to investigate the significance of the model’s unknown parameters in the regulation of GOI expression, we calculated the time integral of the transcription rate within 24 h after RA treatment.

6 $$ \mathrm{Model}\kern0.5em \mathrm{Output}\kern0.5em =\kern0.5em {\int}_0^{24}{\mathrm{I}}_{\max (GOI)}\left(\frac{f_{GOI}\left[\mathrm{RA}:\mathrm{RAR}\right]}{f_{GOI}\left[\mathrm{RA}:\mathrm{RAR}\right]+{k}_{d\left( TF: DNA\right)}}\right)\kern0.5em \mathrm{dt} $$

**Fig. 3:**
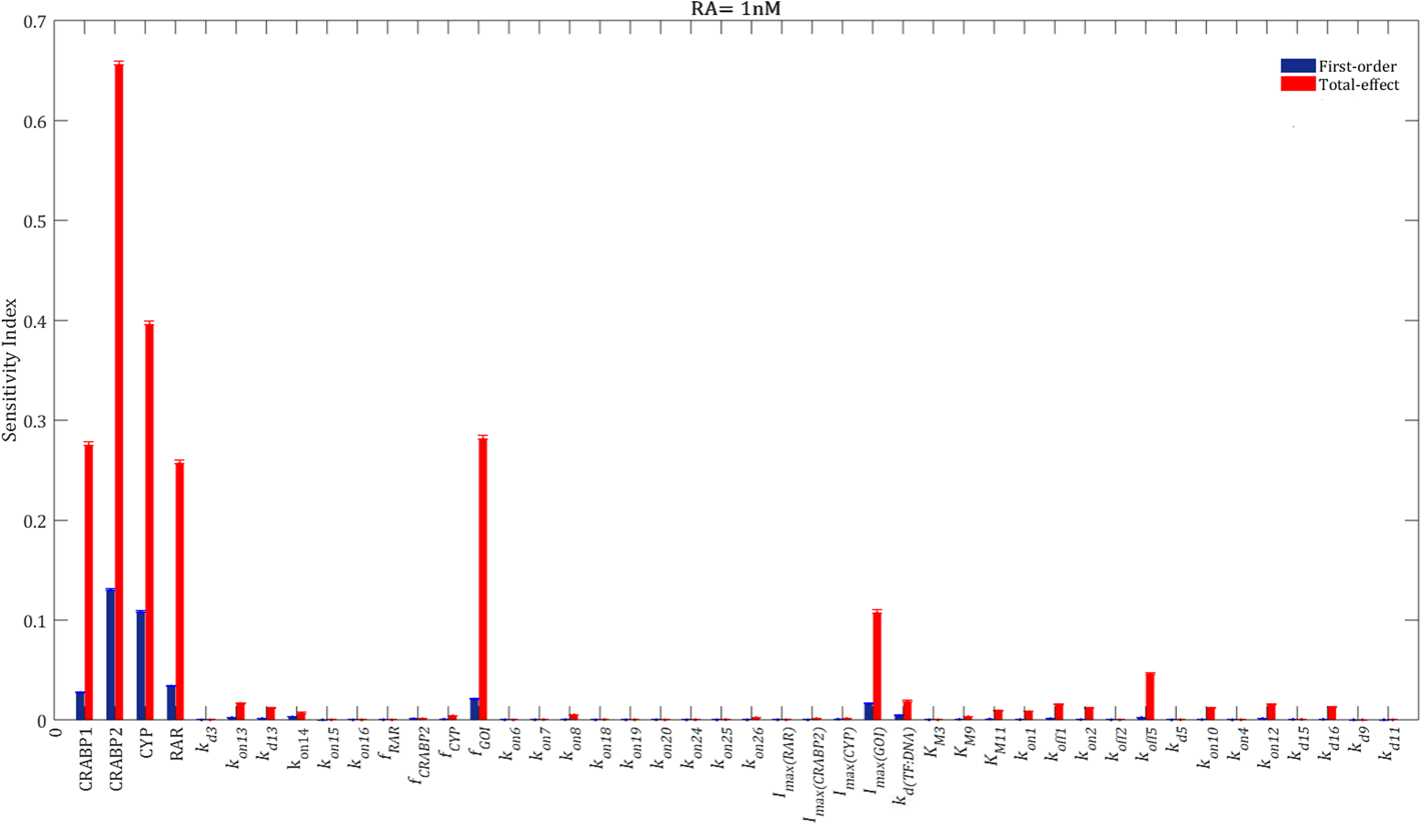
Sensitivity ranking of the model parameters. The model output was set to the time integral of the transcription rate of the GOI within 24 h after adding 1 nM of RA to the model. Blue bars indicate first-order sensitivity indices, while red bars represent total-effect sensitivity indices. The error bars show the bootstrap confidence intervals (95% confidence intervals) of the mean values [36]. Detailed parameter description is provided in Additional file 1

We then calculated the sensitivity of the model output to variations in the model parameters when the cells were treated with 1 nM of RA (Fig. 3). The model parameters, including total concentration of the RA binding proteins, kinetic rate constants, transcription factor fractions and maximal transcription rates were varied within their ranges of variability (full details in Additional file 1).

First-order and total-effect sensitivity indices of the model parameters indicated that the system performance was mainly controlled by the transcription factor fraction of the GOI (*f*_*GOI*_) and the total concentrations of RA binding receptors (Fig. 3). The sensitivity of the output to variations in *f*_*GOI*_ is trivial, since *f*_*GOI*_ represents what portion of RARs can activate the transcription of the GOI.

CRABP2 and RAR were the most and the least important RA receptors controlling RA-mediated mRNA production when RA = 1 nM, respectively. CYP was the second most sensitive parameter in the model, followed by CRABP1. The maximal transcription rate of the GOI (*I*_*max(GOI)*_) and the equilibrium dissociation constant of the transcription factor binding to DNA (*k*_*d(TF:DNA)*_) were other sensitive parameters in the model (Fig. 3). The maximal transcription rate of a given gene can change from cell to cell since the elongation rate of the gene by RNA polymerase can vary across cell lines and across a population of cells of the same type [19]. RA upregulates the expression of the *CRABP2*, *RAR* and *CYP* genes [20]. We modeled these pathways using Eq. 4 with different values of transcription factor fractions and maximal transcription rates, i.e. *f*_*CRABP2*_, *f*_*RAR*_, *f*_*CYP*_, *I*_*max(CRABP2)*_, *I*_*max(RAR)*_, and *I*_*max(CYP)*_ (see Additional file 1). Our results, however, indicated that these pathways did not considerably affect the model output when RA = 1 nM, since the total-effect indices of *f*_*CRABP2*_, *f*_*RAR*_, *f*_*CYP*_, *I*_*max(CRABP2)*_, *I*_*max(RAR)*_, and *I*_*max(CYP)*_ were smaller than 0.01 (Fig. 3).

**Fig. 4:**
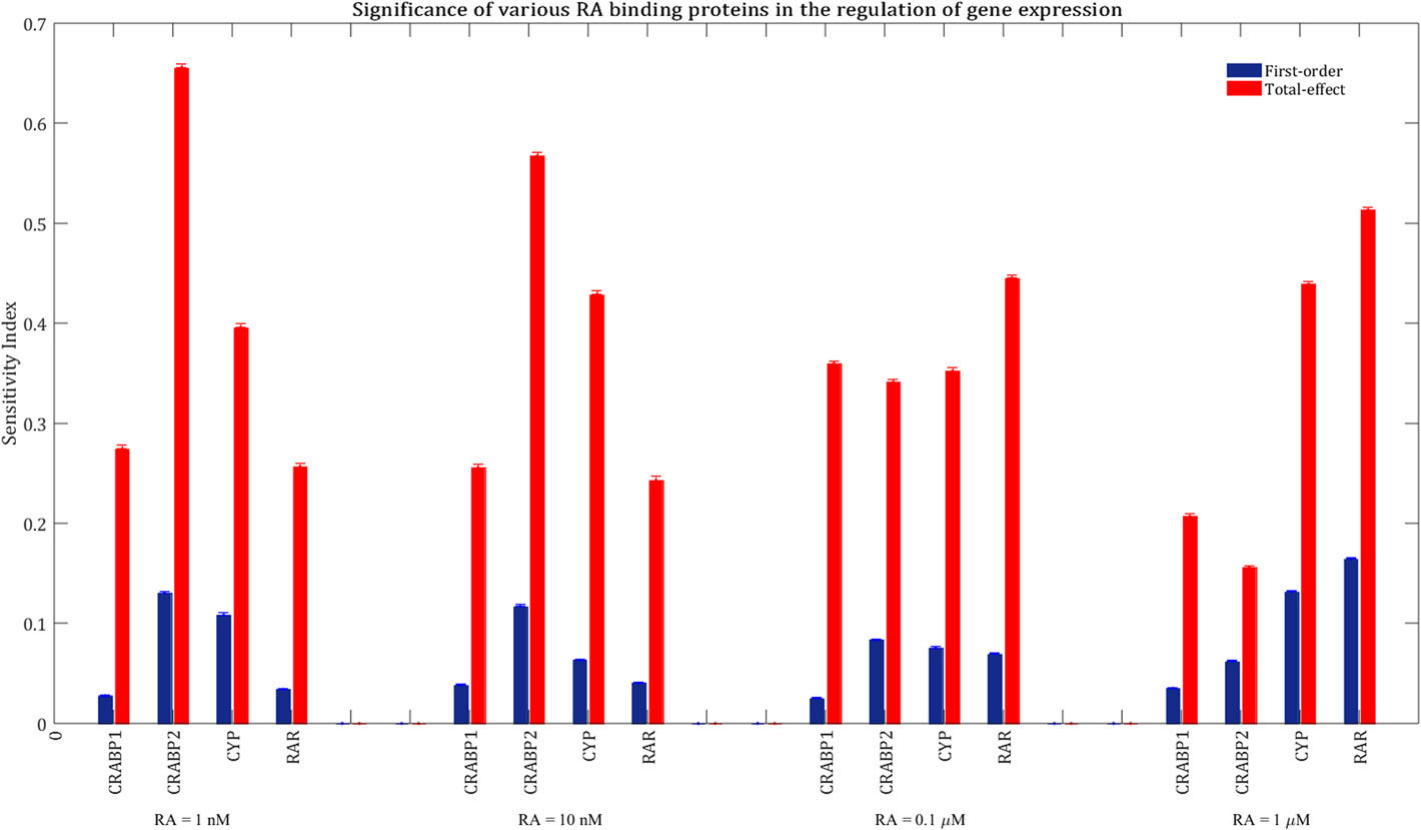
Significance of the RA binding proteins in influencing total mRNA production after treatment with various concentrations of RA. The blue bars show first-order sensitivity indices, while the red bars show total-effect sensitivity indices. The error bars indicate the bootstrap confidence intervals (95% confidence intervals) of the sensitivity indices

We then calculated the sensitivity indices of the model parameters when the model was treated with other concentrations of RA ranging from 10 nM to 1 μM. Our results showed that transcription factor fraction of the GOI, maximal transcription rate of the GOI and total concentration of RA binding receptors mainly controlled the system performance at all concentrations of RA (see Additional file 1: Figure S1). Figure 4 compares the sensitivity indices of RA binding proteins at various concentrations of RA.

RAR is the least important protein in influencing mRNA production when the cells are treated with physiological levels of RA (1–10 nM). That is because RA is mainly bound to CRABP1, CRABP2 and CYP at low concentrations of RA, as those proteins have higher binding affinities than RAR for RA. Thus, variation in total concentration of RAR is less important than variations of the rest of RA binding proteins concentrations in changing the formation rate of RA:RAR, since there are not many free RA molecules available to bind to RARs at physiological levels of RA. However, a change in CRABP2, CRABP1 and CYP concentrations can remarkably accelerate or slow down the transport of RA molecules to RARs, which are mainly unbound at physiological conditions. In other words, RA is the limiting and RAR is the excess species at physiological levels of RA, while RAR is the limiting and RA is the excess species at higher concentrations of RA.

**Fig. 5:**
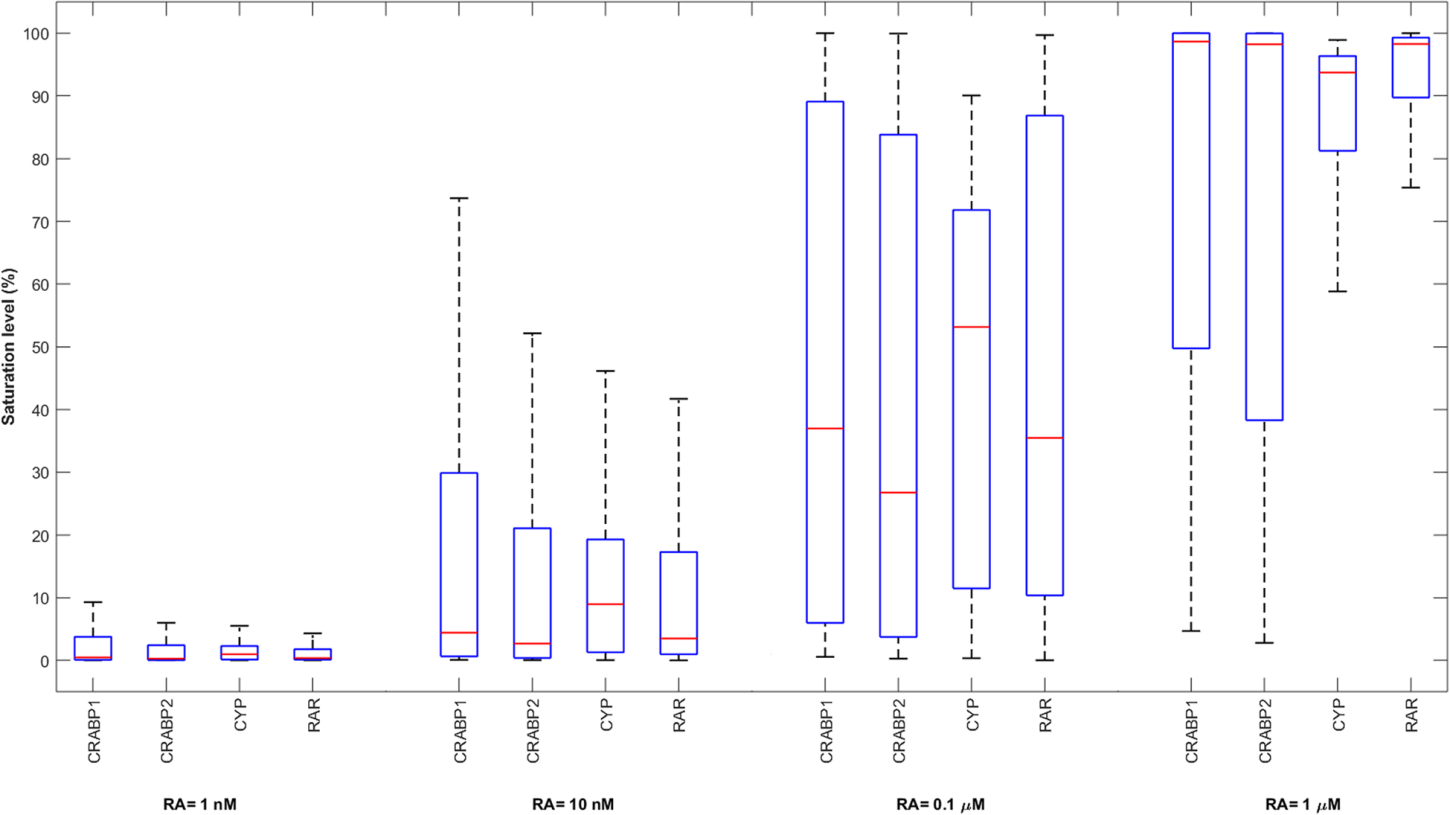
Variation in the saturation index of various RA binding proteins at different concentrations of RA. 10,000 points were randomly sampled, following a uniform distribution over a 44-dimensional parameter space. The models were treated with various concentrations of RA and the saturation indices were calculated

Total concentration of RAR is the most important parameter in influencing mRNA production when RA = 1 μM. This is because RAR is close to saturation with RA at higher levels of RA, since there are more RA molecules accessible to RARs. Thus, enhancement of total RAR concentration can increase the activation rate of the transcription factor, which leads to an increase in the mRNA production rate according to Eq. 4. Figure 5 shows the variations of the RA binding receptors saturation indices at different concentrations of RA. Saturation index of each receptor is defined as the maximum value of the bound fraction of the receptor within 24 h after RA treatment. The bound fraction of a receptor changes over time, and is expressed as:

$$ \mathrm{Bound}\ \mathrm{fraction}\ \mathrm{of}\ \mathrm{a}\ \mathrm{receptor}\kern0.5em =\kern0.5em \frac{\mathrm{Liganded}\ \mathrm{receptor}\ \mathrm{concentration}}{\mathrm{Liganded}\ \mathrm{receptor}\ \mathrm{concentration}+\mathrm{free}\ \mathrm{receptor}\ \mathrm{concentration}} $$

CRABP1 and CRABP2 are less important than RAR when RA = 1 μM, even though they are also close to saturation (Fig. 5). This is because RAR is almost saturated with RA at high levels of RA, and providing RARs with more RA molecules through changing CRABP1 and CRABP2 concentrations does not change the formation rate of activated RAR significantly.

**Fig. 6:**
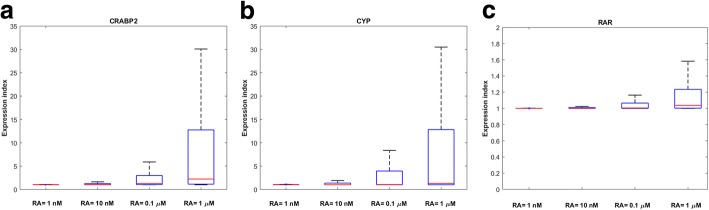
Variation in the expression index of (**a**) CRABP2, (**b**) CYP, (**c**) RAR at various concentrations of RA. 10,000 points were randomly sampled following a uniform distribution over a 44-dimensional parameter space to generate this Figure

Another factor that makes RAR more important than other binding proteins in mRNA production at pharmacological conditions (RA = 0.1–1 μM) is the higher expression rate of CRABP2, RAR and CYP genes at pharmacological levels of RA compared to physiological levels. Total concentrations of RAR, CRABP2 and CYP increase after adding RA to the system. Figure 6 shows the variations in RA binding protein expression indices at different RA concentrations. The expression index of each RA binding protein is defined as the average concentration of each RA binding protein within 24 h after RA treatment, divided by the initial concentration of RA binding protein before the RA therapy. Our results show that the expression indices of all RA binding receptors increase with RA concentration. The expression indices of CRABP2 and CYP are larger than the expression index of RAR at all concentrations of RA. This is because CRABP2 and CYP have larger maximal transcription rates, translation constants, and smaller degradation rates than RAR (see Additional file 1).

Total concentration of CYP contributes almost equally to variations in mRNA production at all concentrations of RA (Fig. 4). This is because CYP level affects the concentrations of free CRABPs available for transferring RA to the nuclear receptors (according to Eqs. 1 to 3).

From Fig. 4, it can be understood that CRABP2 is a more important factor in the RA signaling pathway when the model was treated with physiological levels of RA (1–10 nM) compared to pharmacological levels of RA (0.1–1 μM). This is because RAR is barely saturated with RA at physiological conditions, so that variations in CRABP2 concentration can significantly change the rate of RA transport to RARs.

**Fig. 7:**
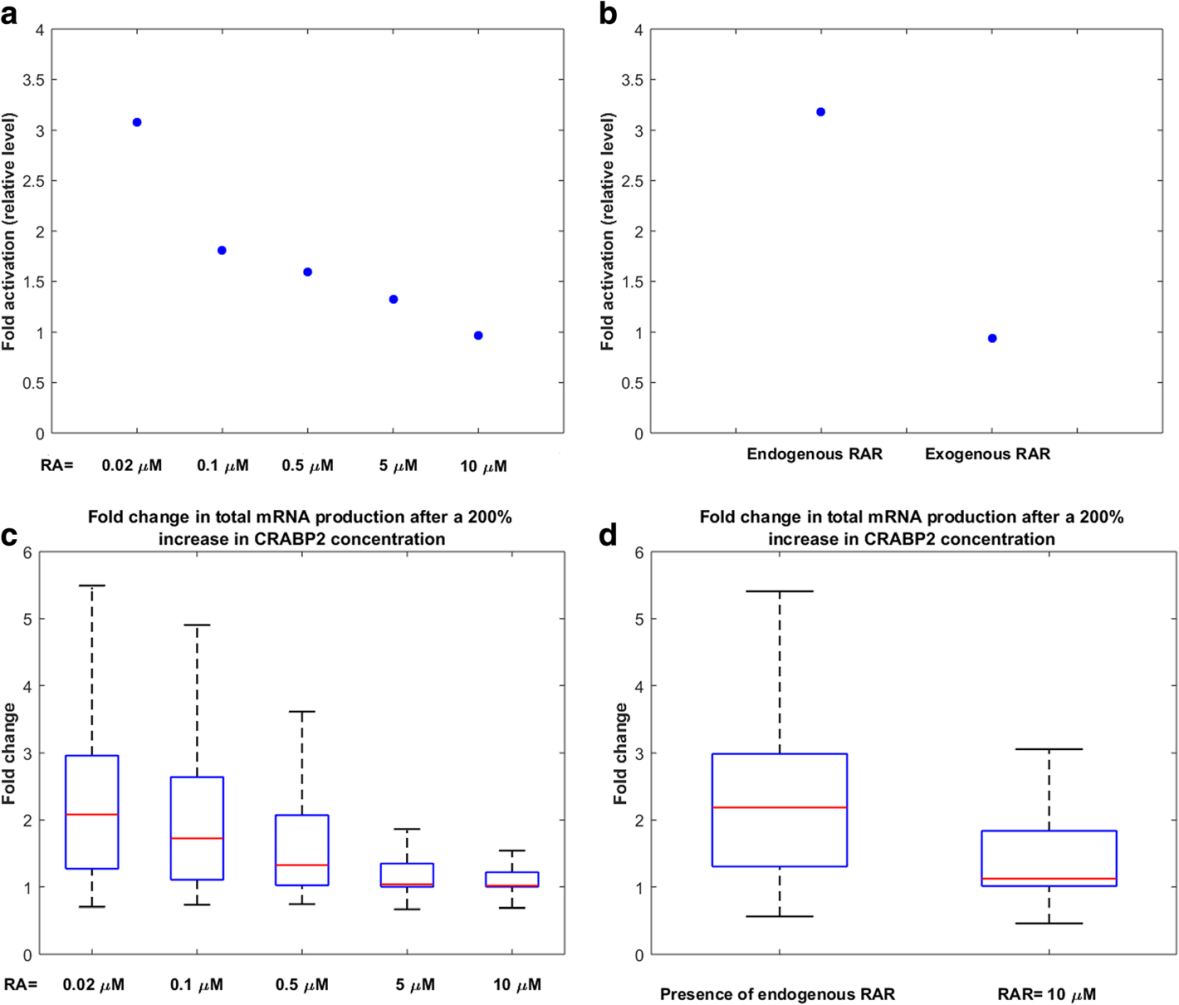
Effects of CRABP2 on transcriptional activity of RAR at various levels of RA and RAR. COS-7 cells were transfected with a luciferase reporter construct driven by a RAR responsive element, and the activity level of the reporter was measured in different conditions. (**a**) Luciferase activity level after adding exogenous levels of CRABP2 to the cells at various concentrations of RA. Data are presented as fold induction relative to luciferase reporter activity level before overexpression of CRABP2. Experimental data was obtained from [7]. (**b**) Luciferase activity level after adding exogenous levels of CRABP2 to the cells, in the presence of endogenous RAR or upon overexpression of RAR. Data are presented as fold induction relative to luciferase reporter activity level before overexpression of CRABP2. Experimental data was given from [7]. (**c**) Fold change in total mRNA production after increasing CRABP2 concentration by 200%. Data are normalized by total mRNA production before CRABP2 overexpression. 10,000 points were randomly sampled following a uniform distribution over a 44-dimensional parameter space, to generate this Figure. (**d**) Fold change in total mRNA production after increasing CRABP2 concentration by 200% in the presence of endogenous RAR or exogenous RAR, i.e. RAR = 10 μM. Data are normalized by total mRNA production before CRABP2 overexpression. 10,000 points were randomly sampled following a uniform distribution over a 44-dimensional parameter space, to generate this Figure

The fact that CRABP2 is more influential in mRNA production at physiological conditions compared to pharmacological conditions is in qualitative accordance with previous experimental studies [7]. A previous in vitro study [7] indicated that exogenous levels of CRABP2 increased the transcriptional activity of RAR only when the concentrations of RA or RAR were limiting (Fig. 7a-b). We performed a local sensitivity analysis to investigate the effect of a constant change in CRABP2 concentration on total mRNA production, over broad regions of RA and RAR concentrations. For this purpose, we sampled several sets of parameters within their ranges of variability, which characterized various cell types or various cells of the same type. We then calculated fold change values of the total mRNA production for each model after increasing CRABP2 concentration by 200% (Fig. 7c). Our results indicated that for a vast majority of cell types, a constant change in CRABP2 concentration is more important in the RA signaling pathway at lower concentrations of RA. We also obtained fold change values of total mRNA production after increasing CRABP2 concentration by 200% in the absence or presence of exogenous levels of RAR (Fig. 7d). Our results showed that variation of CRABP2 concentration is more important at lower concentrations of RAR. This result is in qualitative agreement with experimental observations [7] in COS-7 cells culture (Fig. 7).

The total-effect sensitivity index of CRABP2 is larger than that of CRABP1 when RA = 1–10 nM, while CRABP2 and CRABP1 contribute almost equally to variations in the model output when RA = 0.1–1 μM (Fig. 4). Total-effect sensitivity indices should be used to compare the total contributions of different inputs to variations in the model response. For example, CRABP2 has a larger first-order sensitivity index than CRABP1 when RA concentration is 0.1 or 1 μM, while the total-effect sensitivity index of CRABP1 is slightly larger than that of CRABP2 (Fig. 4). This suggests that CRABP1 interacts stronger than CRABP2 with other parameters.

The calculated sensitivity indices at each RA concentration indicate the relative importance of the parameters at the specified RA concentration. Thus, these indices cannot be used to compare the absolute values of the produced mRNA when the cells were treated with various levels of RA. CRABP2, for instance, has a larger sensitivity index at physiological concentration of RA compared to pharmacological concentrations. However, this does not mean that a constant change in CRABP2 concentration results in a larger variation in molar production of the mRNA at physiological levels of RA compared to the pharmacological concentration of RA. In general, with a fixed set of values for RA binding receptor concentrations, total mRNA production increases by RA dose.

### RA degradation pathway

RA metabolism is crucial in RA signaling not only because the CYP can limit the amount of RA available to interact with RARs, but also because some RA metabolites can induce the transcription of some target genes through specific pathways [21, 22]. Furthermore, RA resistance, observed in continuous RA treatment in cancer patients, is at least in part due to RA degradation. RA metabolism is mediated mainly by CYP enzymes, which are found in different cell types. Even though several studies have investigated the role of various families of CYP in biosynthesis of RA, little is known about the contribution of CRABPs and RARs in the RA degradation pathway. In this section we investigated the contributions of the RA binding receptors to production of RA metabolites. In our model, RA was only degraded via CYP enzymes, while interacting with CYP directly or indirectly. In the direct mechanism, free RA molecules can bind to CYP, while the indirect process involves CRABP1 and CRABP2 as carrier proteins that transfer RA to CYP. Thus, the total rate of RA degradation is obtained by

7 $$ \frac{\mathrm{d}\left[\mathrm{RA}\kern0.5em \mathrm{metabolites}\right]}{\mathrm{d}\mathrm{t}}=\kern0.5em {k}_{on4}\left[\mathrm{RA}:\mathrm{CYP}\right]+\kern0.5em {k}_{on10}\left[\mathrm{RA}:\mathrm{CRABP}1:\mathrm{CYP}\right]+\kern0.5em {k}_{on12}\left[\mathrm{RA}:\mathrm{CRABP}2:\mathrm{CYP}\right] $$

where *k*_*on4*_*k*_*on10*_ and *k*_*on12*_ are degradation rate constants of RA : CYP, RA : CRABP1 : CYP and RA : CRABP2 : CYP, respectively.

We performed GSA to investigate the sensitivity of total RA metabolite production within 24 h after RA therapy, to variations in the model’s unknown parameters.

8 $$ \mathrm{Model}\kern0.5em \mathrm{Output}\kern0.5em =\kern0.5em {\int}_0^{24}{k}_{on4}\left[\mathrm{RA}:\mathrm{CYP}\right]+\kern0.5em {k}_{on10}\left[\mathrm{RA}:\mathrm{CRABP}1:\mathrm{CYP}\right]+\kern0.5em {k}_{on12}\left[\mathrm{RA}:\mathrm{CRABP}2:\mathrm{CYP}\right]\kern0.5em \mathrm{dt} $$

**Fig. 8:**
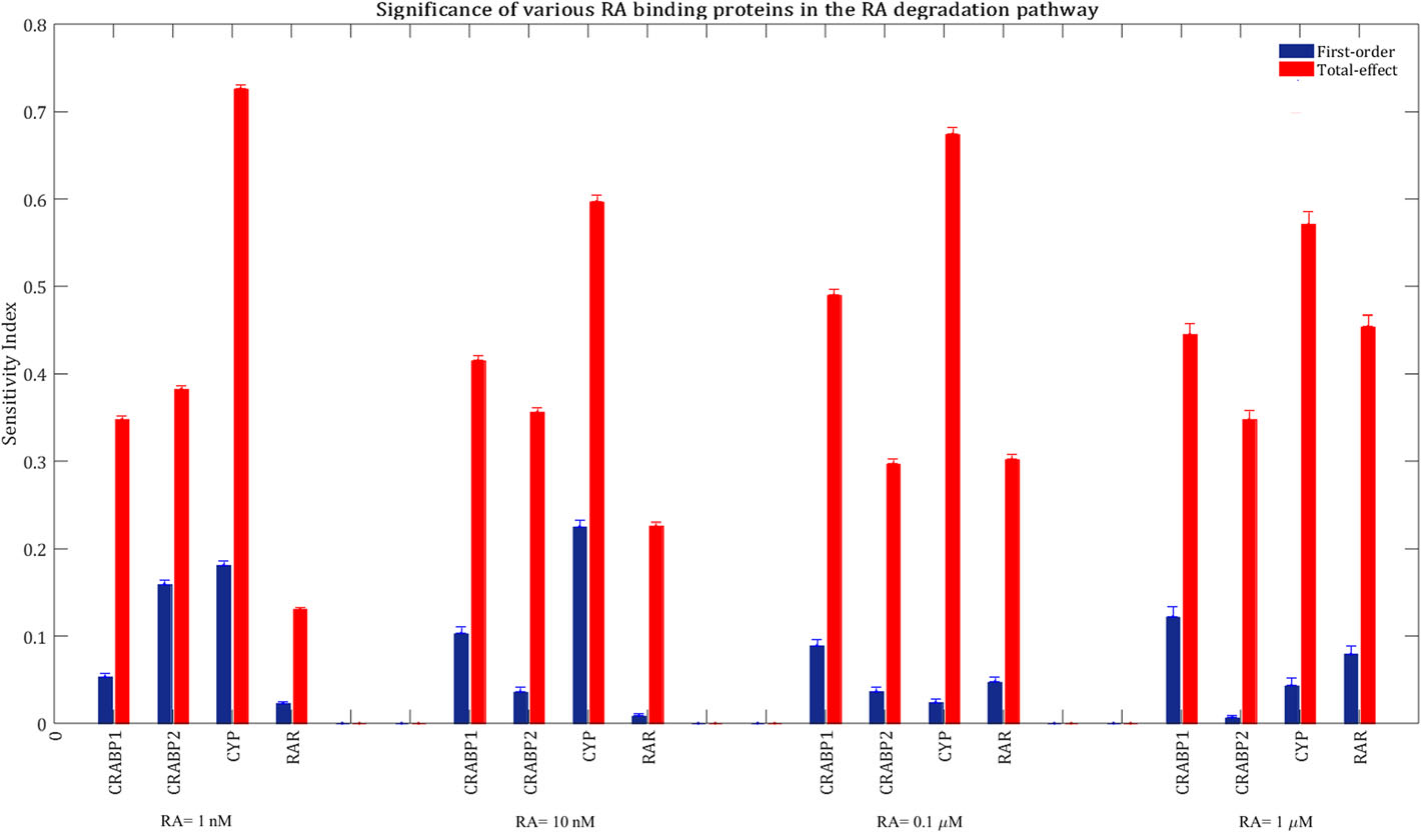
Relative importance of various RA binding proteins in total RA metabolite formation at various concentrations of RA. The model output was set to total RA metabolite formation within 24 h of treatment with various concentrations of RA. The blue bars show first-order sensitivity indices, while the red bars show total-effect sensitivity indices. The error bars indicate the bootstrap confidence intervals (95% confidence intervals) of the sensitivity indices

As in the previous section, we considered physiological bounds for the parameters and used the Sobol’s method to calculate the sensitivity indices. Our results showed that the production of RA metabolites was mainly affected by cellular concentrations of the RA binding proteins (Additional file 1: Figure S2). CYP had the largest total-effect sensitivity index at all RA concentrations, which shows that total concentration of CYP was the most important parameter controlling the system performance (Fig. 8). CRABP1 and CRABP2 contribute almost equally to variation in the model response when RA = 1–10 nM, while CRABP1 is more important than CRABP2 in the RA degradation pathway when RA = 0.01–1 μM.

RAR becomes more important in the RA degradation pathway as RA concentration increases. This can be explained by the fact that at high concentrations of RA, RAR is the most important parameter that controls RA-induced gene expression (Fig. 4). The cellular level of RAR can significantly influence RA-induced upregulation of CYP, CRABP2 and RAR. Our results indicated that RA-induced upregulation of CYP had significant effects on total RA metabolite formation when RA = 0.01–1 μM (see Additional file 1: Figure S2). From Fig. 8, it can be understood that for a given RA concentration, the rank order of first-order sensitivity indices of the parameters is not necessarily the same as the rank order of total-effect sensitivity indices. This is due to different levels of interaction of each parameter with the rest of the parameters. Furthermore, our results were obtained using GSA, which gives some insights into the functions of various receptors by covering the entire parameter space. However, it might be possible that for a specific set of initial concentrations the rank order of parameter sensitivities would be different.

**Fig. 9:**
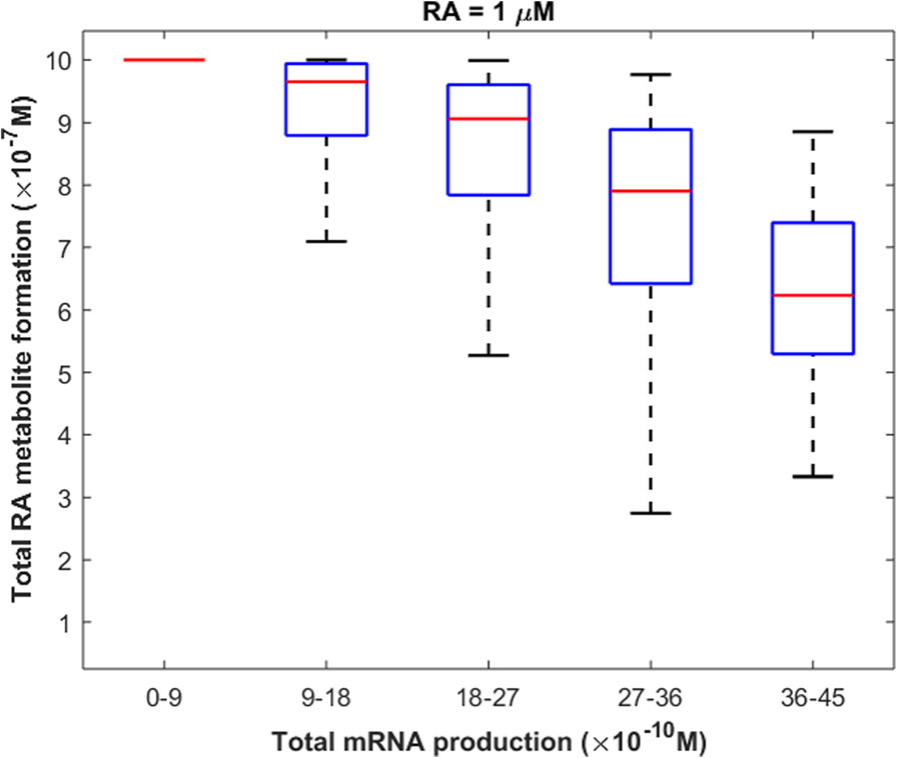
The relationship between total RA metabolite formation and total mRNA production within 24 h of treatment with 1 μM of RA. 10,000 points were randomly sampled following a uniform distribution over a 44-dimensional parameter space

Comparing Fig. 4 with Fig. 8, one can observe that for a given RA concentration, the rank order of sensitivity of the RA binding receptors is not the same for total mRNA production and total RA metabolite formation. To further investigate the relationship between mRNA production and RA metabolite formation by the RA signaling pathway, we calculated Spearman’s rank correlation coefficient between total mRNA production and total RA product formation within 24 h of treatment with 1 μM of RA. Our results revealed a significant negative correlation (ρ = − 0.7, *p* = 0, *n* = 10,000) between total mRNA production and total RA metabolite formation (Fig. 9). However, Spearman’s rank correlation coefficient decreased with the reduction of RA concentration (Additional file 1: Figure S3).

**Fig. 10:**
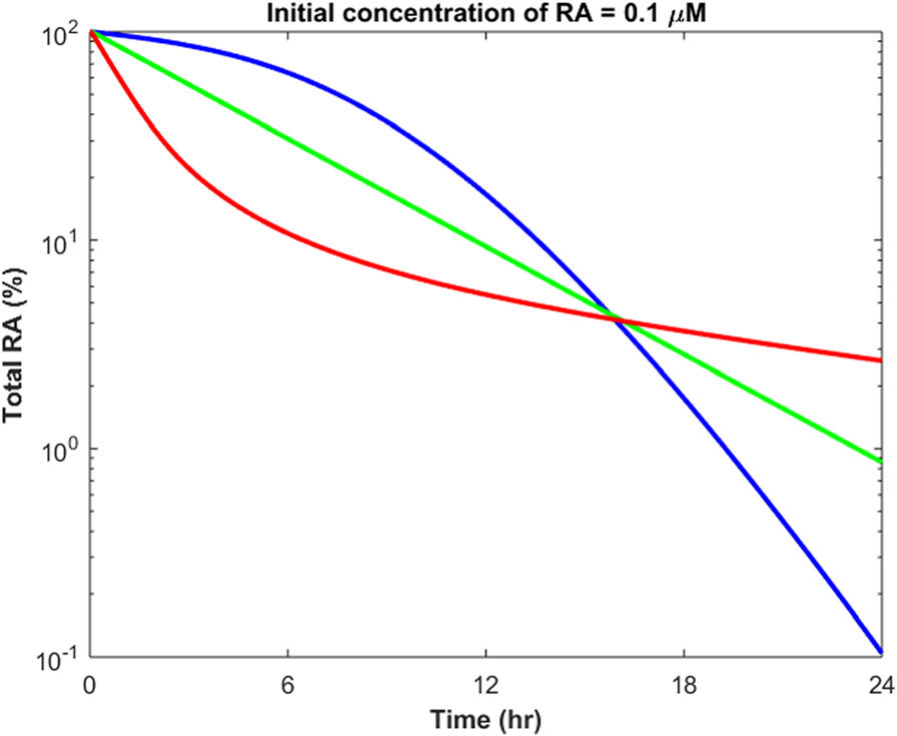
Various forms of elimination of RA after treating different models with 0.1 μM of RA. Three models with various parameter sets are shown in blue, green and red. Green: [CRABP1_*t*_]=8.6 μM, [CRABP2_*t*_]=16.1 nM, [CYP_*t*_]=11.2 nM, [RAR_*t*_]=0.24 μM. Red: [CRABP1_*t*_]=2.7 nM, [CRABP2_*t*_]=11.4 nM, [CYP_*t*_]=30 nM, [RAR_*t*_]=0.22 μM. Blue: [CRABP1_*t*_]=3 μM, [CRABP2_*t*_]=11 nM, [CYP_*t*_]=2.26 nM, [RAR_*t*_]=0.71 μM. Full list of the models’ parameters is reported in Additional file 1: Table S2

One serious drawback of the clinical use of RA is that RA has a rapid and variable degradation rate [23, 24]. Thus, a relatively high concentration of RA is required to induce the expression of target genes in various cell types. The pattern of RA degradation is important since it can directly influence cell differentiation and gene expression by RA. In this section, we simulated the variations in total concentration of RA within 24 h after RA treatment. For this purpose, we sampled several sets of parameters within their ranges of variability, which characterized various cell types or various cells of the same type. We then added 0.1 μM of RA to each model and obtained the changes in total RA concentration over time. Our results showed that RA exhibited different elimination patterns depending on intracellular concentrations of the RA binding proteins, i.e. CRABP1, CRABP2, CYP and RAR (Fig. 10). Furthermore, RA can both down- and up-regulate its own degradation.

### Effects of the RA binding proteins on the efficacy and toxicity of RA

**Fig. 11:**
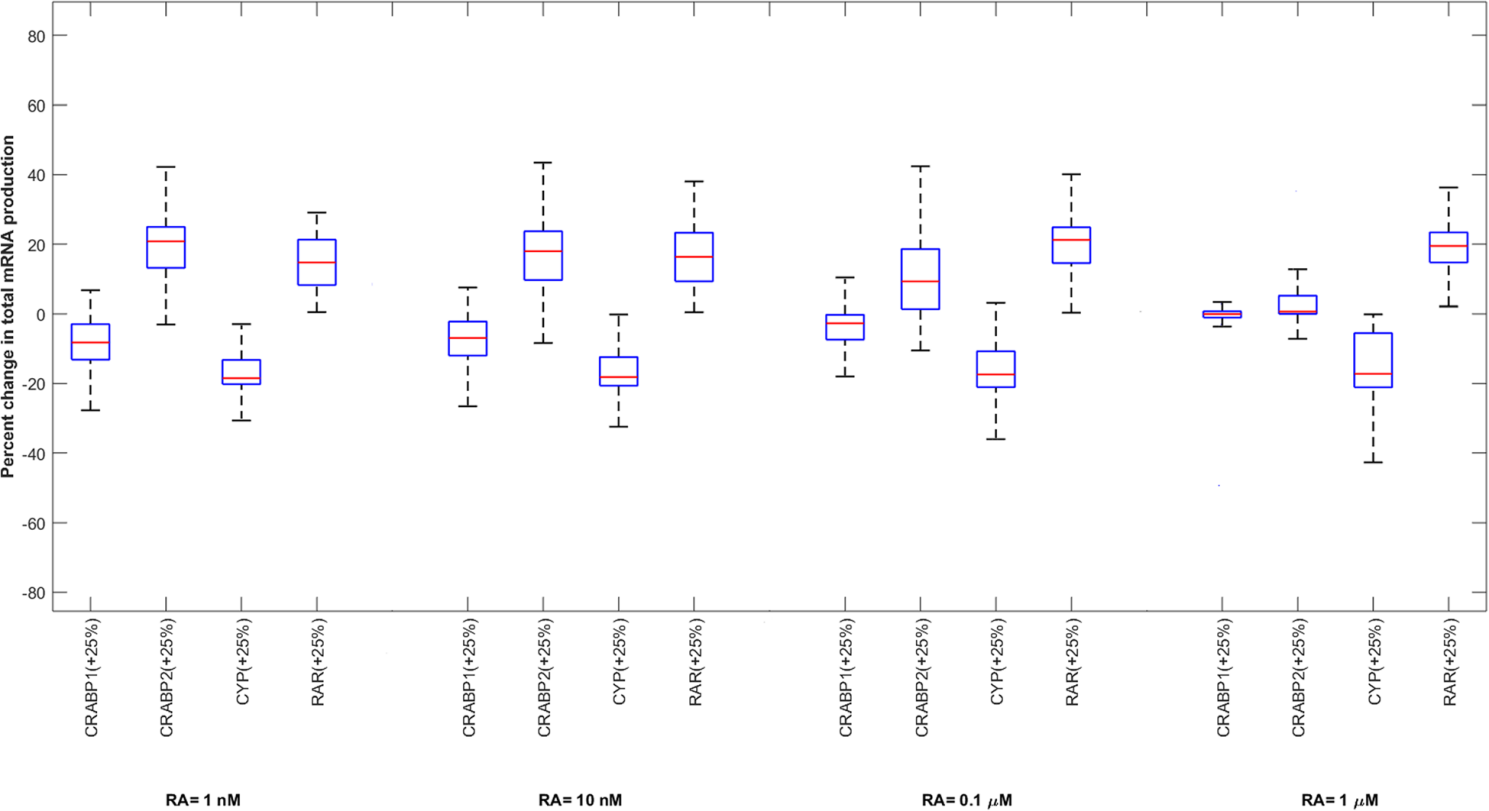
Variation in total mRNA production after a 25% increase in each RA binding protein concentration, while the rest of the parameters are constant. 10,000 points were randomly sampled following a uniform distribution over a 44-dimensional parameter space, to generate this Figure

An understanding of the roles and significance of RA binding proteins in the RA signaling pathway is important for both therapeutic and toxicological reasons. The results presented in this study can be used to develop pharmacological methods to increase the maximal response produced by RA. These pharmacological approaches can vary depending on cancer type, as different cell types have different expression levels of RA binding proteins. For example, in pharmacological conditions (RA = 1 μM), induction of expression of the *RAR* gene or inhibition of expression of the *CYP* gene have more significant effects than overexpression of the *CRABP2* gene on the expression levels of the GOI in a given cell type (Fig. 4). To further investigate how the total mRNA production at various RA concentrations is sensitive to variation in each RA binding protein concentration, we performed a local sensitivity analysis. In this regard, 10,000 sets of parameters were randomly sampled, following a uniform distribution over a 44-dimensional parameter space. Variation in the total mRNA production was calculated for each model after increasing the concentration of each RA binding protein by 25% while the rest of the parameters remained unchanged (Fig. 11).

Our results indicate that a 25% increase in CRABP1 or CRABP2 concentrations is more important at physiological concentrations of RA compared to pharmacological concentrations, which is in accordance with our global sensitivity analysis results (Fig. 4). CRABP2 is the most influential protein at physiological conditions, while RAR and CYP are the most important proteins when RA = 1 μM. From Fig. 11, it can be understood that a 25% increase in the total concentration of RAR enhances mRNA production for all models. A 25% increase in total concentration of either CYP or CRABP1 decreases total mRNA production for most of the models, while a 25% increase in CRABP2 concentration enhances total mRNA production for the majority of models. In general, the way that the variation in total concentrations of CRABP1, CRABP2 or CYP affects mRNA production depends on the cellular concentrations of all RA binding receptors. Overexpression of CRABP1, for example, can increase or decrease the transcriptional activity of the target gene, depending on total concentrations of the other RA receptors. This is because these proteins complex with each other in the absence or presence of RA.

The results presented in this paper can provide insight into the efficacy and safety of RA therapy in treatment of different cancer types and cancer patients. CRABP1, CRABP2, CYP and RAR expressions can be upregulated or downregulated depending on the cancer type [25–28] and administrated anticancer drugs [29, 30]. Cancer patients usually take different medications at the same time. Concurrent use of other drugs with RA can influence the RA signaling pathway in at least two ways. First, interaction with other medicines can cause variations in the pharmacokinetics and pharmacodynamics of RA, significantly changing its efficacy and toxicity. For instance, it is possible that two or more drugs compete for the same CYP enzyme in a cancer cell, since CYP-mediated metabolism is a major route of elimination for many drugs. This competitive inhibition can decrease the availability of CYP enzymes to RA, therefore decreasing its metabolism rate and increasing its toxicity. Second, some drugs can inhibit or induce the expression of RA binding proteins such as CYP [29, 30]. Variations in the concentrations of RA binding proteins may affect the efficacy of RA over the course of cancer therapy. For instance, CRABP1 and CRABP2 are the least important parameters in the model when RA = 1 μM (Fig. 4). Thus, up-regulation or down-regulation of these proteins due to other factors such as disease progress, drug interactions, etc. should not change the rate of mRNA production by RA significantly. However, if for example use of a strong RAR inhibitor or CYP inducer is unavoidable for the patient, the therapeutic effects of RA may be decreased significantly.

## Discussion

Retinoic acid, a metabolite of vitamin A, modulates a wide variety of biological processes such as cell growth, cell differentiation and cell proliferation. RA has also been known to be effective in treatment of various types of cancer. Even though a vast number of studies have focused on exploring the regulatory target genes for RA, the significance and roles of various intracellular RA receptors in transduction of the RA signal have not been fully understood. CRABP1, CRABP2, CYP enzymes and RARs are the main intracellular proteins which can bind to RA as receptors. Few previous studies have attempted to investigate the effects of overexpression of CRABPs on the RA signaling pathway, and in some cases somewhat contradictory results have been reported for different cell lines [2–4]. In this study, we developed a mathematical model to analyze the importance of CRABP1, CRABP2, CYP and RAR in production of mRNA and RA metabolites. In this regard, after proposing a well-mixed model of the RA signaling pathway, we performed a global sensitivity analysis to investigate the relative importance of RA binding receptors in total mRNA production via the RA pathway. Our results indicate that CRABP2 is the most important RA receptor at physiological levels of RA, while RAR concentration has the least importance among all four RA receptors. At pharmacological levels of RA, the total mRNA production was more sensitive to variations in RAR and CYP levels than CRABP1 and CRABP2 levels. It is important to note that all RA binding receptors could influence RA-induced mRNA production within the entire region of parameter space where the concentrations of RA binding proteins change considerably. They are all important since their sensitivity indices were of the same order of magnitude. These results can explain the conflict between previous experimental results regarding the effects of CRABP1 on transcriptional activity of target genes [1, 3, 4]. Our results were obtained using GSA, which quantifies the effects of the model inputs on the model output by perturbing the inputs within large ranges. Therefore, our results indicate that in a broader region of parameter space, which represents various cells with various levels of RA receptors, all of the RA binding receptors are influential. However, there is a possibility that for a certain parameter set which specifies a specific tissue or cell, CRABP1 is unimportant in the RA pathway. Thus, for a given cell type, an accurate parameter set is necessary to determine whether a parameter has a substantial control on the system performance.

Our local sensitivity analysis indicated that CRABP2 is more important in the RA signaling pathway at lower concentrations of RA or RAR. This result is in qualitative agreement with in vitro observations in COS-7 cells [7]. Our model can be applied to various cell types and our results can be validated experimentally once more information is available about the expression levels of RA binding proteins in the cell types of interest.

Our GSA analysis indicated that RAR-mediated increases in CRABP2 and CYP concentrations after RA therapy were more important in the regulation of GOI expression than the RAR-mediated increase in RAR concentration (see Additional file 1: Figure S1). This is because total-effect sensitivity indices of *I*_*max(CRABP2)*_ and *I*_*max(CYP)*_ were larger than total-effect sensitivity index of *I*_*max(RAR)*_. Furthermore, total-effect sensitivity indices of *f*_*CRABP2*_ and *f*_*CYP*_ were larger than total-effect sensitivity index of *f*_*RAR*_ at all concentrations of RA (Additional file 1: Figure S1). The time-dependent increases of CRABP2, CYP and RAR concentrations after RA therapy can alter the relative concentrations of RA binding proteins. Thus, RA receptors can become increasingly or decreasingly important in the RA signaling pathway as time goes on. In this study, we calculated the sensitivity of the model’s outputs, i.e. total mRNA production and total RA metabolite formation, to variations in total concentrations of the RA binding receptors before RA treatment. Thus, the significance of RAR-mediated upregulation of *CRABP2, CYP* and *RAR* genes in the RA signaling pathway is mainly shown by the sensitivity index of total RAR concentration, since RAR is the only RA receptor mediating the transcription of target genes.

This study has some limitations. First, we assumed that RA influences gene expression through the classical pathway, which involves binding of RA to a nuclear hormone receptor heterodimer (RAR:RXR). The liganded heterodimer can initiate the transcription of target genes after binding to a DNA response element. However, there may be other intermediate transcription factors or nonclassical pathways that can transduce RA signal, thus our results can only be applied to the genes which are direct targets of the classical RA signaling pathway. Second, we assumed that all RA binding proteins undergo first-order degradation processes. This may not be the case for all types of tissues with various expression levels of degradation enzymes. The mechanisms mediating the elimination of RA binding receptors have not been fully understood, thus the model can be improved once more information regarding these mechanisms is available. Third, we used the kinetic rate constants of CYP26B1 in the model. CYP26B1 is a member of the 26 family (CYP26s) of the CYP enzymes which is mainly responsible for metabolism of RA during adult life [6, 31–33]. However, RA can also be degraded by other families of CYP which are different from CYP26B1 in terms of rate constants and binding affinities. In the current model, we assumed that the kinetic rate constants of degrading enzymes can vary by a factor of two around the in vitro values for CYP26B1. This assumption increases the applicability of our results to other cell types with different types of CYP. Thus, our results are applicable to those cell lines that express higher levels of CYP26B1 compared to other CYP families and to those cell types which have CYP enzymes with kinetic rate constants within the specified ranges in this study. The current simulation can be run using the kinetic rate constants of any arbitrary CYP enzyme. In that case, this model can be expanded to include the effects of RA metabolites on RA-induced gene expression if the CYP of interest forms high levels of active RA metabolites. The current model is applicable to those cell types whose main degrading enzyme is CYP26B1. The primary metabolite formed by CYP26B1 from RA is 4-OH-RA [34, 35]. CYP26B1 forms non-bioactive dehydroxylated products from 4-OH-RA [35]. Thus, we believe that the endogenous levels of RA metabolites formed by CYP26B1 do not play significant roles in the RA signaling pathway. However, there are other active RA metabolites such as 4-oxo-RA which can potentially compete with RA for binding to RAR and activating the transcription of target genes [22]. Fourth, we neglected the possible effects of RA treatment on the model parameters such as translation rate constants, transcription rate constants, and degradation rate constants of proteins and mRNAs. Fifth, for simplicity, we proposed a well-mixed model, thus our model is not able to capture the dynamics of protein diffusion through the nuclear membrane. RARs are located inside the cell nucleus. RA must diffuse across the nucleus membrane to be able to bind to RARs. In reality, RA binds to CRABPs after diffusing across the cellular membrane. RA can diffuse across the nuclear membrane alone or bound to CRABPs. We believe that our well-mixed model can approximate this process due to the rank order of binding affinity of RA for various RA receptors. RA binds to CRABP1 and CRABP2 with higher affinity than to RAR, which implies that RA is primarily available for CRABPs. The remaining RA molecules can bind to RARs and CYP enzymes. Finally, we assumed that the ratio of total transcription factor concentration to total RAR concentration (*f*) remains constant after adding RA to the cell. However, this depends on the gene- and cell-type. It is believed that RARs and RXRs each have three isotypes, namely RAR_α_, RAR_β_, RAR_γ_, RXR_α_, RXR_β_, RXR_γ_, which can form nine different heterodimers. Depending on the gene-type, one or some of these heterodimers can initiate the transcription of the target gene after binding to RA. Little is known about the expression levels of the nuclear hormone receptors in various cell types, and their interactions with each other. The model presented in this paper can be expanded once there is more information about the nuclear hormone receptor expression levels and functions.

## Conclusions

Cellular levels of retinoic acid receptor (RAR), cytochrome P450 (CYP) enzymes and cellular retinoic acid binding proteins (CRABP1 and CRABP2) significantly affect the rate of gene expression through the classical retinoic acid (RA) signaling pathway. In this study, we used computational modeling to investigate the significance of various RA binding proteins in the regulation of expression of a gene of interest (GOI) under physiological or pharmacological conditions. A better understanding of the roles and significance of RA binding proteins in the RA signaling pathway could lead to the development of pharmacological methods to induce or block the activity of specific RA binding receptor (s), thereby improving the efficacy of the RA. Our results indicate that CRABP2 and CYP concentrations are more influential than CRABP1 and RAR concentrations in controlling mRNA production by the RA signaling pathway in physiological concentrations of RA (1–10 nM). However, RAR is the most sensitive parameter of the model in pharmacological conditions (RA = 0.1–1 μM). We also identified the critical proteins in the RA metabolism pathway, and showed that there is a significant negative correlation between RA-induced mRNA production and RA metabolite formation after 24 h of treatment with 1 μM of RA. Our results demonstrate that the pattern of RA degradation following RA therapy depends on the cell type.

## Additional file

Additional file 1: Detailed model description and supplementary results. (DOCX 1863 kb)

## Abbreviations

CRABP1: Cellular retinoic acid binding protein 1
CRABP2: Cellular retinoic acid binding protein 2
CYP: Cytochrome P450
GOI: Gene of interest
GSA: Global sensitivity analysis
ODE: Ordinary differential eq.
RA: Retinoic acid
RAR: Retinoic acid receptor
RARE: Retinoic acid response element
RXR: Retinoid X receptor
