# Supplementary material for: Identification of influential proteins in the classical retinoic acid signaling pathway
Source: Theor Biol Med Model. 2018 Oct 16;15:16. doi: 10.1186/s12976-018-0088-7 (PMC6190658; doi:10.1186/s12976-018-0088-7)
Supplement: Supplementary file 1 — Detailed model description and supplementary results. (DOCX 1863 kb) [file 12976_2018_88_MOESM1_ESM.docx]

**Supplementary Information**

**Formulation of the model**

The model described how RA is eliminated from the cell by CYP enzymes. CYP enzymes can metabolize not only free RA but also RA molecules bound to CRABP1 and CRABP2 [1]. RA degradation was modeled by Michaelis-Menten kinetics with *in vitro* enzyme kinetics parameters

$$E+S\overset{\Leftrightarrow}{k_{d}}\mathrm{ES}\underset{\Rightarrow}{k_{cat}}E+Product,$$

$$K_{M}=\frac{k_{off}+k_{cat}}{k_{on}} ,$$

$$k_{d}=\frac{k_{off}}{k_{off}} ,$$

where $K_{M}$ and $k_{d}$ are the Michaelis constant and the equilibrium dissociation constant of the enzyme substrate complex. Formation of products from RA:CRABP1 had a $k_{d}$ of 0.024 nM and a $K_{M}$ of 21.7 nM, while formation of products from RA:CRABP2 had a $k_{d}$ of 0.059 nM and a $K_{M}$ of 24.3 nM [1]. The $K_{M}$ value was set at 64.6 nM for the interaction between CYP and free RA, while the equilibrium dissociation constant of RA:CYP complex was unknown. Formation of RA metabolites had catalytic rate constants of 10.2, 16.8 and 27 $1/hr$ from RA:CRABP1, RA:CRABP2 and free RA, respectively [1].

Most of the cellular impacts of RA rely on variation in gene expression. The expression of a gene of interest (GOI) is initiated once the activated transcription factor ($\mathrm{TF}_{a}$) binds to DNA at a retinoic acid response element (RARE). RAR/RXR heterodimer is the main transcription factor in the classical RA signaling pathway, and becomes activated once it binds to RA. The transcription rate of a target gene depends on the promoter occupancy. The binding of an activated transcription factor to a promoter can be described by

$$\mathrm{TF}_{a}+\mathrm{Promoter}\overset{\Leftrightarrow}{k_{d(TF:DNA)}}\mathrm{TF}_{a}:\mathrm{Promoter}$$

where $k_{d(TF:DNA)}$ is the equilibrium dissociation constant of the transcription factor binding to the promoter, and ranges from 15 to 33 nM [2]. The fraction of time that any given promoter spends in the transcription factor-bound state is given by [3-5]

$$Fraction of binding time=\frac{[\mathrm{TF}_{a}]}{[\mathrm{TF}_{a}]+k_{d(TF:DNA)}} . (S1)$$

The rate of the gene transcription is proportional to the fraction of the binding time,

$$\frac{I}{I_{\max(GOI)}}=\frac{[\mathrm{TF}_{a}]}{\left[ \mathrm{TF}_{a} \right]+k_{d(TF:DNA)}} , (S2)$$

where $I_{max(GOI)}$is the maximal transcription rate of the GOI by the activated transcription factor ($\mathrm{TF}_{a}$). The value of $I_{max(GOI)}$ depends on gene-type, cell-type and the transcription factor [6]. RA can regulate the expression of *RAR*, *CRABP2* and *CYP* genes [7]. The values of $I_{max(RAR)}$, $I_{max(CRABP2)}$ and $I_{max(CYP)}$ were set at ${4.1\times10}^{-11}$*,* ${1.5\times10}^{-10},$ and ${1.06\times10}^{-10} \frac{M}{\mathrm{hr}}$, respectively. These values were obtained by dividing the average elongation rate of RNA polymerase by the gene lengths. The average elongation rate for RNA polymerase is $2 \frac{\mathrm{kbp}}{\min}$ [8], while the lengths of *RAR*, *CRABP2* and *CYP* genes are 48.4 kbp (chr 17, GRCh38.p7), 13.4 kbp (chr 1, GRCh38.p7) and 18.6 kbp (chr 2, GRCh38.p7), respectively [9-11].

Assuming that RA binds to various RAR isoforms with the same binding affinity, the ratio of the activated transcription factor ($\mathrm{TF}_{a}$) concentration to the total transcription factor ($\mathrm{TF}_{t}$) concentration is the same as the ratio of the liganded RAR concentration to total RAR concentration, thus

$$\frac{[\mathrm{TF}_{a}]}{[\mathrm{TF}_{t}]}=\frac{[RA:RAR]}{[\mathrm{RAR}_{t}]} (S3)$$

The concentration of total transcription factor which can activate the transcription of the GOI is a portion of the concentration of total RAR,

$$f_{GOI}=\frac{[\mathrm{TF}_{t}]}{{[RAR}_{t}]} (S4)$$

where $f_{GOI}$ represents the transcription factor fraction of the GOI with a value between 0 and 1. Transcription factor fraction is cell-dependent for a given gene. The rate of mRNA production via the RA pathway is obtained by combining Eqs. S2 to S4.

$$\frac{I}{I_{max(GOI)}}=\frac{f_{GOI}[RA:RAR]}{f_{GOI}\left[ RA:RAR \right]+k_{d(TF:DNA)}} . (S5)$$

It is important to note that we assumed that the association/dissociation between DNA and transcription factor is in equilibrium because it occurs much faster than the other binding/unbinding reactions in the model [3, 12-14].

In our model, proteins and mRNAs are degraded by first-order reactions. Species are degraded in both bound and unbound forms. For example, CRABP1:CYP may undergo either CRABP1 degradation or CYP degradation.

*CRABP2*, *CYP* and *RAR* genes had both basal transcription rate ($I_{0}$) and RA-induced transcription rate (Eq. S5), while CRABP1 had a constant rate of protein expression, since *CRABP1* gene is not a target for RA [7, 15].

The full set of reactions and rate constants in the current model are shown in Table 2 and Table S1, respectively.

We then modeled the chemical reactions by ODEs.

$$\frac{d[RA]}{\mathrm{dt}}=-k_{on1}\left[ \mathrm{RA} \right]\left[ CRABP1 \right]+k_{off1}\left[ RA:CRABP1 \right]-k_{on2}\left[ \mathrm{RA} \right]\left[ CRABP2 \right]+k_{off2}\left[ RA:CRABP2 \right]-k_{on3}\left[ \mathrm{RA} \right]\left[ \mathrm{CYP} \right]+k_{off3}\left[ RA:CYP \right]-k_{on5}\left[ \mathrm{RA} \right]\left[ \mathrm{RAR} \right]+k_{off5}\left[ RA:RAR \right]+k_{on21}\left[ RA:CRABP1 \right]+k_{on22}\left[ RA:CRABP2 \right]+k_{on23}\left[ RA:RAR \right] (S6)$$

$$\frac{d[CRABP1]}{\mathrm{dt}}=-k_{on1}\left[ \mathrm{RA} \right]\left[ CRABP1 \right]+k_{off1}\left[ RA:CRABP1 \right]-k_{on17}\left[ CRABP1 \right]+K-k_{on15}\left[ \mathrm{CYP} \right]\left[ CRABP1 \right]+k_{off15}\left[ CYP:CRABP1 \right]+k_{off28}\left[ CYP:CRABP1 \right] (S7)$$

$$\frac{d[RA:CRABP1]}{\mathrm{dt}}=k_{on1}\left[ \mathrm{RA} \right]\left[ CRABP1 \right]-k_{off1}\left[ RA:CRABP1 \right]-k_{on21}\left[ RA:CRABP1 \right]-k_{on9}\left[ \mathrm{CYP} \right]\left[ RA:CRABP1 \right]+k_{off9}\left[ RA:CRABP1:CYP \right] (S8)$$

$$\frac{d[CRABP2]}{\mathrm{dt}}={-k}_{on2}\left[ \mathrm{RA} \right]\left[ CRABP2 \right]+k_{off2}\left[ RA:CRABP2 \right]+k_{on7}\left[ {CRABP2}_{\mathrm{mRNA}} \right]{-k}_{on16}\left[ \mathrm{CYP} \right]\left[ CRABP2 \right]+k_{off16}\left[ CYP:CRABP2 \right]+k_{on30}\left[ CYP:CRABP2 \right]+k_{on14}\left[ RA:CRABP2:RAR \right]-k_{on18}\left[ CRABP2 \right] (S9)$$

$$\frac{d[RA:CRABP2]}{\mathrm{dt}}=k_{on2}\left[ \mathrm{RA} \right]\left[ CRABP2 \right]-k_{off2}\left[ RA:CRABP2 \right]-k_{on22}\left[ RA:CRABP2 \right]-k_{on13}\left[ RA:CRABP2 \right]\left[ \mathrm{RAR} \right]+k_{off13}\left[ RA:CRABP2:RAR \right]-k_{on11}\left[ RA:CRABP2 \right]\left[ \mathrm{CYP} \right]+k_{off11}\left[ RA:CRABP2:CYP \right] (S10)$$

$$\frac{d[CYP]}{\mathrm{dt}}=-k_{on15}\left[ \mathrm{CYP} \right]\left[ CRABP1 \right]+k_{off15}\left[ CYP:CRABP1 \right]{-k}_{on16}\left[ \mathrm{CYP} \right]\left[ CRABP2 \right]+k_{off16}\left[ CYP:CRABP2 \right]+k_{on8}\left[ {CYP}_{\mathrm{mRNA}} \right]-k_{on9}\left[ \mathrm{CYP} \right]\left[ RA:CRABP1 \right]+k_{off9}\left[ RA:CYP:CRABP1 \right]-k_{on11}\left[ RA:CRABP2 \right]\left[ \mathrm{CYP} \right]+k_{off11}\left[ RA:CRABP2:CYP \right]+k_{on27}\left[ CRABP1:CYP \right]+k_{on29}\left[ CRABP2:CYP \right]-k_{on19}\left[ \mathrm{CYP} \right]+k_{on4}\left[ RA:CYP \right]-k_{on3}\left[ \mathrm{RA} \right]\left[ \mathrm{CYP} \right]+k_{off3}\left[ RA:CYP \right] (S11)$$

$$\frac{d[RA:CYP]}{\mathrm{dt}}=k_{on3}\left[ \mathrm{RA} \right]\left[ \mathrm{CYP} \right]-k_{off3}\left[ RA:CYP \right]-k_{on4}\left[ RA:CYP \right] (S12)$$

$$\frac{d[RAR]}{\mathrm{dt}}=-k_{on5}\left[ \mathrm{RA} \right]\left[ \mathrm{RAR} \right]+k_{off5}\left[ RA:RAR \right]-k_{on20}\left[ \mathrm{RAR} \right]-k_{on13}\left[ RA:CRABP2 \right]\left[ \mathrm{RAR} \right]+k_{off13}\left[ RA:CRABP2:RAR \right]+k_{on6}\left[ {RAR}_{\mathrm{mRNA}} \right] (S13)$$

$$\frac{d[RA:RAR]}{\mathrm{dt}}=k_{on5}\left[ \mathrm{RA} \right]\left[ \mathrm{RAR} \right]-k_{off5}\left[ RA:RAR \right]+k_{on14}\left[ RA:CRABP2:RAR \right]-k_{on23}\left[ RA:RAR \right] (S14)$$

$$\frac{d[{RAR}_{\mathrm{mRNA}}]}{\mathrm{dt}}=I_{0(RAR)}+I_{max(RAR)}\left( \frac{f_{RAR}\left[ RA:RAR \right]}{f_{RAR}\left[ RA:RAR \right]+k_{d(TF:DNA)}} \right)-k_{on24}\left[ {RAR}_{\mathrm{mRNA}} \right] (S15)$$

$$\frac{d[{CRABP2}_{\mathrm{mRNA}}]}{\mathrm{dt}}=I_{0(CRABP2)}+I_{max(CRABP2)}\left( \frac{f_{CRABP2}\left[ RA:RAR \right]}{f_{CRABP2}\left[ RA:RAR \right]+k_{d(TF:DNA)}} \right)-k_{on25}\left[ {CRABP2}_{\mathrm{mRNA}} \right] (S16)$$

$$\frac{d[{CYP}_{\mathrm{mRNA}}]}{\mathrm{dt}}=I_{0(CYP)}+I_{max(CYP)}\left( \frac{f_{CYP}\left[ RA:RAR \right]}{f_{CYP}\left[ RA:RAR \right]+k_{d(TF:DNA)}} \right)-k_{on26}[{CYP}_{\mathrm{mRNA}}] (S17)$$

$$\frac{d[RA:CRABP1:CYP]}{\mathrm{dt}}=k_{on9}\left[ \mathrm{CYP} \right]\left[ RA:CRABP1 \right]-k_{off9}\left[ RA:CRABP1:CYP \right]-k_{on10}\left[ RA:CRABP1:CYP \right] (S18)$$

$$\frac{d[RA:CRABP2:RAR]}{\mathrm{dt}}=k_{on13}\left[ RA:CRABP2 \right]\left[ \mathrm{RAR} \right]-k_{off13}\left[ RA:CRABP2:RAR \right]-k_{on14}\left[ RA:CRABP2:RAR \right] (S19)$$

$$\frac{d[RA:CRABP2:CYP]}{\mathrm{dt}}=k_{on11}\left[ \mathrm{CYP} \right]\left[ RA:CRABP2 \right]-k_{off11}\left[ RA:CRABP2:CYP \right]-k_{on12}\left[ RA:CRABP2:CYP \right] (S20)$$

$$\frac{d[CRABP1:CYP]}{\mathrm{dt}}=k_{on15}\left[ \mathrm{CYP} \right]\left[ CRABP1 \right]-k_{off15}\left[ CRABP1:CYP \right]-k_{on27}\left[ CRABP1:CYP \right]-k_{on28}\left[ CRABP1:CYP \right]+k_{on10}\left[ RA:CRABP1:CYP \right] (S21)$$

$$\frac{d[CRABP2:CYP]}{\mathrm{dt}}=k_{on16}\left[ \mathrm{CYP} \right]\left[ CRABP2 \right]-k_{off16}\left[ CRABP2:CYP \right]-k_{on29}\left[ CRABP2:CYP \right]-k_{on30}\left[ CRABP2:CYP \right]+k_{on12}\left[ RA:CRABP2:CYP \right] (S22)$$

where the numbers in the subscripts of the rate constants refer to the index of the reactions (Table 2).

The model had 14 independent unknown parameters including unknown rate constants, unknown initial concentrations and unknown transcription factor fractions (Main Text, Table 3). We considered some physiological bounds for the unknown parameters. We also assumed that the values of maximal transcription rate constants, translation rate constants, forward and reverse rate constants, equilibrium dissociation rate constants, Michaelis constants, catalytic rate constants and elimination rates of proteins and mRNAs varied within a factor of two around the *in vitro* values. Overall, our model had 44 independent parameters varying within their ranges.

**Uncertainty Analysis of the Model**

The independent parameters were sampled uniformly over their range of possible values (number of samples=10000). The rest of the unknown parameters (Table S1), and the unknown initial concentrations (Main Text, Table 1) were obtained using the independent parameters. To do so, we assumed that the system was at steady state prior to RA treatment. After obtaining all the parameters, the model was used to obtain total mRNA production within 24 hours of RA treatment. We then used a GSA technique to assess the sensitivity of the model output to variations in model inputs.

**Global Sensitivity Analysis Techniques**

In this study, we used a MATLAB toolbox for global sensitivity analysis, called SAFE [16]. We ranked the model’s parameters in terms of their importance using Sobol’s method, which has been shown to be one of the most effective GSA methods for determining individual and cooperative sensitivities [17-19]. Sobol’s method is a variance-based sensitivity analysis approach that uses the principle of variance decomposition to obtain the sensitivity of each parameter. Given an integrable function $f$ over a *k*-dimensional parameter space $Ω^{k}$,

$y=f\left( x_{1}, x_{2},\ldots, x_{k} \right) (S23)$

Sobol’s method decomposes the response into a set of functions of increasing dimensionality,

$$f\left( x \right)=f_{0}+\sum_{i=1}^{k} f_{i}+\sum_{i=1}^{k} \sum_{j>i}^{k} f_{ij}+\ldots+f_{123\ldots k}, (S24)$$

where each individual term is a function of the parameters in its index. The unconditional variance of the output $V\left( Y \right)$ is defined as

$$V\left( Y \right)=\int_{Ω^{k}} f^{2}\left( x \right) dx-{(\int_{Ω^{k}} f\left( x \right) dx)}^{2}. (S25)$$

The total variance is decomposed into partial variances using the expansion of $f$ into terms of increasing dimensions (Eq. S24).

$$V\left( Y \right)=\sum_{i=1}^{k} V_{i}\left( Y \right)+\sum_{i=1}^{k} \sum_{j>i}^{k} V_{ij}\left( Y \right)+\ldots+V_{123\ldots k}\left( Y \right) . (S26)$$

Based on Sobol’s method, the first-order sensitivity index for each parameter is calculated by

$$S_{i}=\frac{V_{i}\left( Y \right)}{V\left( Y \right)} , (S27)$$

where $V_{i}\left( Y \right)$ is the fraction of the total variance, which is related to changes in the parameter $x_{i}$ over its range of variability. The Sobol total-effect index for the parameter $x_{i}$ is obtained by the sum of all sensitivity indices which have $i$ in their index

$$S_{Ti}=S_{i}+\sum_{i\neq j} S_{ij}+\sum_{i\neq j,i\neq l,j<l} S_{ijl}+\ldots(S28)$$

The total-effect indices account for total contribution of the input to response variation. Total-effect indices can be used to determine the noninfluential parameters in the model. $x_{i}$ can be fixed anywhere within its range of uncertainty if $S_{Ti}=0.$ However, previous studies have shown that parameters with total-effect indices smaller than 0.01 can be considered noninfluential [20-22].

Table S1. Reaction rate constants

|  | $\boldsymbol{k}_{\boldsymbol{on}}$ | | | $\boldsymbol{k}_{\boldsymbol{off}}$ | | |
| --- | --- | --- | --- | --- | --- | --- |
| **Number** | **Range** | **Unit** | **Reference** | **Range** | **Unit** | **Reference** |
| 1 | 2.13e11 | $M^{-1}\mathrm{hr}^{-1}$ | [23] | 13.2 | $\mathrm{hr}^{-1}$ | [23] |
| 2 | 1.85e11 | $M^{-1}\mathrm{hr}^{-1}$ | [23] | 25.2 | $\mathrm{hr}^{-1}$ | [23] |
| 3 | Unknown | $M^{-1}\mathrm{hr}^{-1}$ |  | Unknown | $\mathrm{hr}^{-1}$ |  |
| 4 | 27 | $\mathrm{hr}^{-1}$ | [1] | - |  |  |
| 5 | Unknown^*^ | $M^{-1}\mathrm{hr}^{-1}$ |  | 36-140 | $\mathrm{hr}^{-1}$ | [24] |
| 6 | 135.6 | $\mathrm{hr}^{-1}$ | [6] | - |  |  |
| 7 | 870.7 | $\mathrm{hr}^{-1}$ | [6] | - |  |  |
| 8 | 190.4 | $\mathrm{hr}^{-1}$ | [6] | - |  |  |
| 9 | Unknown | $M^{-1}\mathrm{hr}^{-1}$ |  | Unknown | $\mathrm{hr}^{-1}$ |  |
| 10 | 10.2 | $\mathrm{hr}^{-1}$ | [1] | - |  |  |
| 11 | Unknown | $M^{-1}\mathrm{hr}^{-1}$ |  | Unknown | $\mathrm{hr}^{-1}$ |  |
| 12 | 16.8 | $\mathrm{hr}^{-1}$ | [1] | - |  |  |
| 13 | Unknown | $M^{-1}\mathrm{hr}^{-1}$ |  | Unknown | $\mathrm{hr}^{-1}$ |  |
| 14 | Unknown | $\mathrm{hr}^{-1}$ |  | - |  |  |
| 15 | Unknown | $M^{-1}\mathrm{hr}^{-1}$ |  | Unknown | $\mathrm{hr}^{-1}$ |  |
| 16 | Unknown | $M^{-1}\mathrm{hr}^{-1}$ |  | Unknown | $\mathrm{hr}^{-1}$ |  |
| 17 | 0.0385 | $\mathrm{hr}^{-1}$ | [25] | - |  |  |
| 18 | 0.0385 | $\mathrm{hr}^{-1}$ | [25] | - |  |  |
| 19 | 0.00835 | $\mathrm{hr}^{-1}$ | [6] | - |  |  |
| 20 | 0.173 | $\mathrm{hr}^{-1}$ | [26] | - |  |  |
| 21 | 0.0385 | $\mathrm{hr}^{-1}$ | [25] | - |  |  |
| 22 | 0.0385 | $\mathrm{hr}^{-1}$ | [25] | - |  |  |
| 23 | 0.173 | $\mathrm{hr}^{-1}$ | [26] | - |  |  |
| 24 | 0.138 | $\mathrm{hr}^{-1}$ | [27] | - |  |  |
| 25 | 0.0347 | $\mathrm{hr}^{-1}$ | [28] | - |  |  |
| 26 | 0.11 | $\mathrm{hr}^{-1}$ | [6] | - |  |  |
| 27 | 0.0385 | $\mathrm{hr}^{-1}$ | [25] | - |  |  |
| 28 | 0.00835 | $\mathrm{hr}^{-1}$ | [6] | - |  |  |
| 29 | 0.0385 | $\mathrm{hr}^{-1}$ | [25] | - |  |  |
| 30 | 0.00835 | $\mathrm{hr}^{-1}$ | [6] | - |  |  |

- Forward rate constant of reaction 5 is obtained using an equilibrium dissociation constant between 6 and 20 nM [13, 24].

Table S2. Randomly sampled parameters of the models shown in Figure 10.

| **Parameters** | **Blue curve** | **Green curve** | **Red curve** |
| --- | --- | --- | --- |
| $CRABP1$ | 2.99E-06 | 8.60E-06 | 2.73E-09 |
| $CRABP2$ | 1.09E-08 | 1.61E-08 | 1.14E-08 |
| $\mathrm{CYP}$ | 2.26E-09 | 1.12E-08 | 2.91E-08 |
| $\mathrm{RAR}$ | 7.08E-07 | 2.36E-07 | 2.22E-07 |
| $k_{d3}$ | 1.72E-08 | 2.68E-08 | 2.45E-08 |
| $k_{on13}$ | 4.25E+09 | 1.2E+10 | 2E+10 |
| $k_{d13}$ | 7.88E-10 | 1.60E-10 | 1.84E-10 |
| $k_{on14}$ | 152.592 | 74.9655 | 72.16303 |
| $k_{on15}$ | 2.58E+10 | 1.85E+10 | 1.96E+10 |
| $k_{on16}$ | 7.51E+09 | 2.96E+10 | 3.46E+10 |
| $f_{RAR}$ | 0.790299 | 0.083393 | 0.332411 |
| $f_{CRABP2}$ | 0.116596 | 0.543783 | 0.287017 |
| $f_{CYP}$ | 0.826228 | 0.558422 | 0.03116 |
| $f_{GOI}$ | 0.592287 | 0.978981 | 0.404229 |
| $k_{on6}$ | 103.5768 | 75.87115 | 71.91922 |
| $k_{on7}$ | 439.5742 | 962.9376 | 978.0601 |
| $k_{on8}$ | 145.5691 | 276.7654 | 234.8322 |
| $k_{on18}$ | 0.067334 | 0.051427 | 0.049882 |
| $k_{on19}$ | 0.005092 | 0.005032 | 0.014976 |
| $k_{on20}$ | 0.227853 | 0.125652 | 0.149364 |
| $k_{on24}$ | 0.245181 | 0.074328 | 0.119889 |
| $k_{on25}$ | 0.049549 | 0.027726 | 0.049374 |
| $k_{on26}$ | 0.146517 | 0.154336 | 0.12501 |
| $I_{max(RAR)}$ | 7.17E-11 | 6.33E-11 | 3.20E-11 |
| $I_{max(CRABP2)}$ | 2.61E-10 | 8.20E-11 | 7.56E-11 |
| $I_{max(CYP)}$ | 1.37E-10 | 1.99E-10 | 9.54E-11 |
| $I_{max(GOI)}$ | 0.780444 | 1.926296 | 1.293431 |
| $k_{d(TF:DNA)}$ | 2.10E-08 | 2.70E-08 | 1.64E-08 |
| $K_{M3}$ | 1.20E-07 | 9.41E-08 | 5.53E-08 |
| $K_{M9}$ | 1.55E-08 | 1.15E-08 | 4.23E-08 |
| $K_{M11}$ | 3.86E-08 | 2.79E-08 | 4.81E-08 |
| $k_{on1}$ | 1.99E+11 | 2.51E+11 | 2.46E+11 |
| $k_{off1}$ | 18.31229 | 22.79166 | 20.27421 |
| $k_{on2}$ | 3.22E+11 | 2.62E+11 | 1.03E+11 |
| $k_{off2}$ | 22.66635 | 29.69411 | 14.14157 |
| $k_{off5}$ | 98.29141 | 76.1627 | 117.3805 |
| $k_{d5}$ | 1.01E-08 | 1.66E-08 | 8.89E-09 |
| $k_{on10}$ | 20.04989 | 17.52512 | 19.15578 |
| $k_{on4}$ | 35.2612 | 49.85688 | 15.49563 |
| $k_{on12}$ | 19.36441 | 18.11537 | 8.855135 |
| $k_{d15}$ | 8.16E-08 | 8.86E-08 | 8.18E-08 |
| $k_{d16}$ | 4.64E-08 | 7.50E-08 | 9.29E-08 |
| $k_{d9}$ | 1.57E-11 | 4.00E-11 | 3.07E-11 |
| $k_{d11}$ | 1.08E-10 | 7.91E-11 | 7.74E-11 |


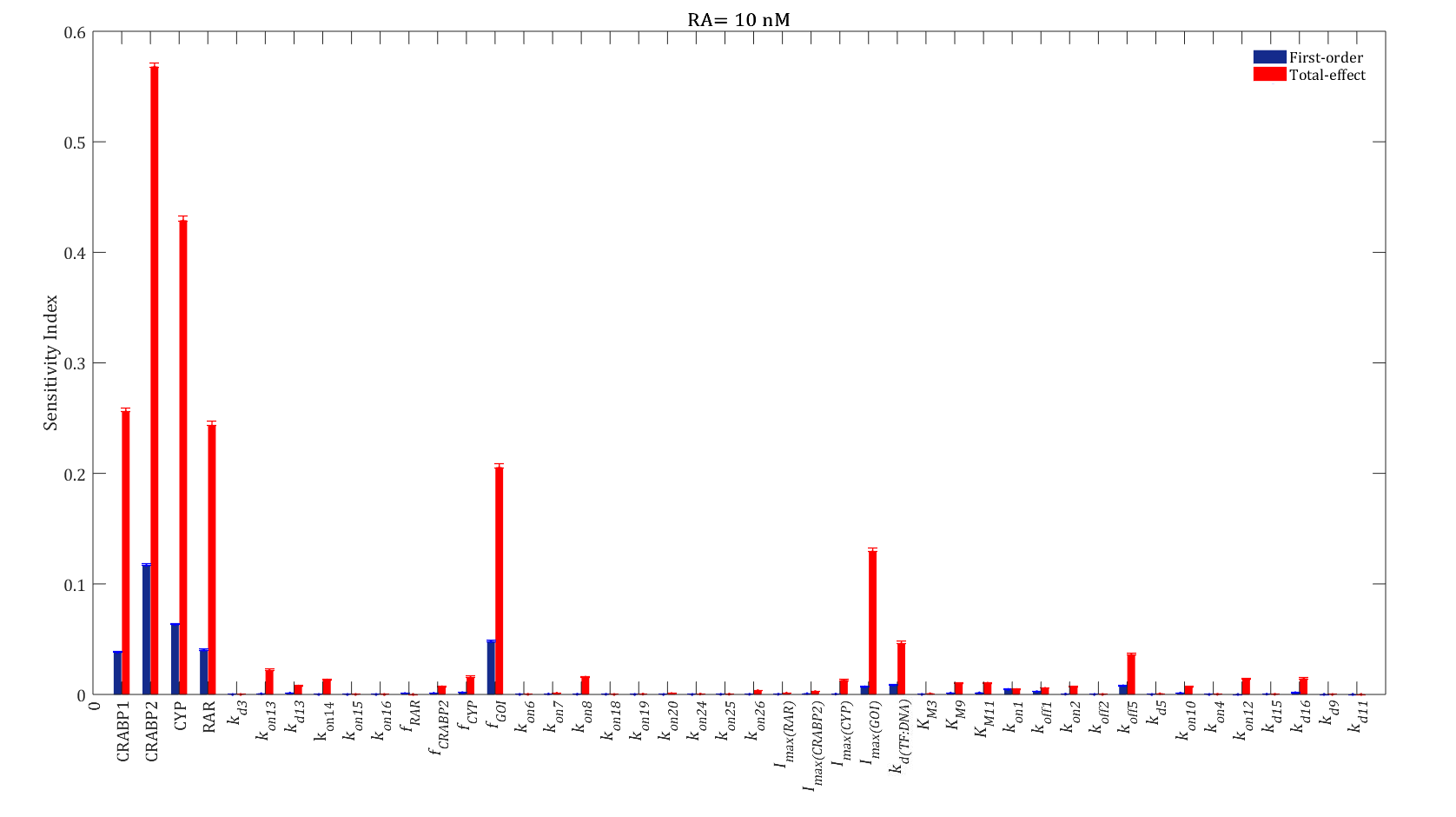


Figure S1. (a)


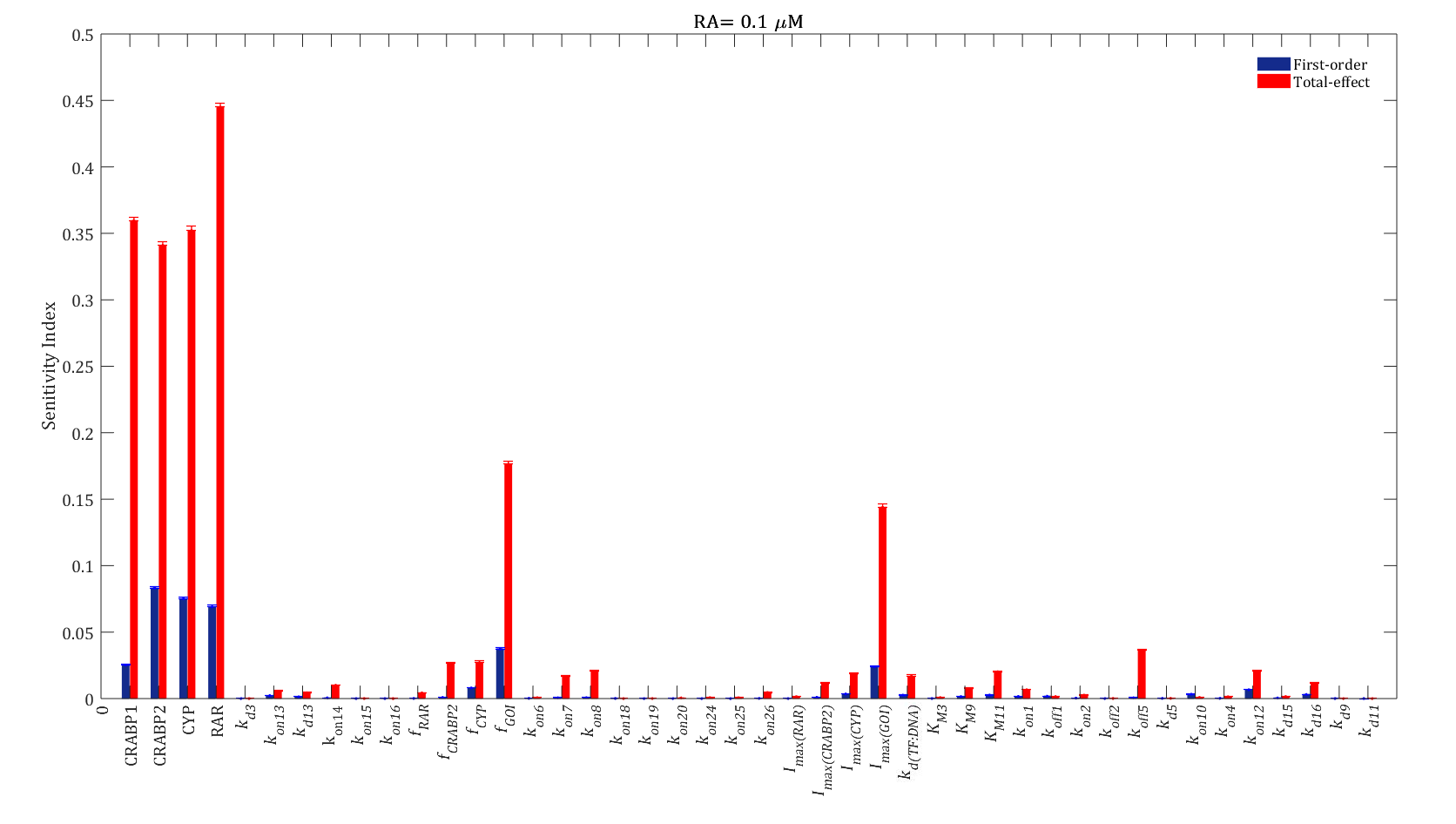


Figure S1. (b)


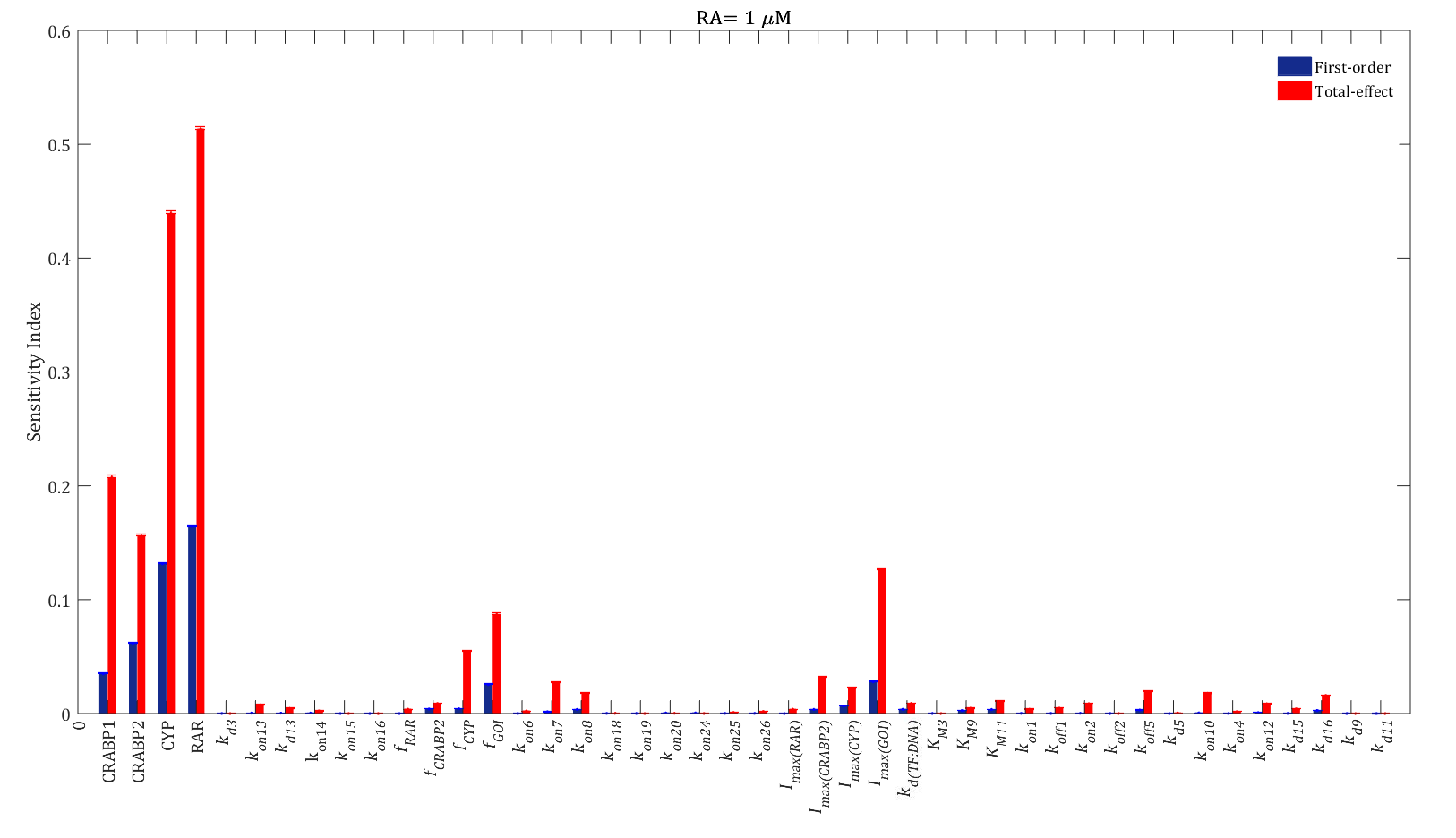


Figure S1. (c)

Figure S1. Sensitivity ranking of the model parameters. The model output was set to the time integral of the transcription rate of the GOI within 24 hours after adding (a) 10 nM, (b) 0.1 µM and (c) 1 µM of RA to the model. Blue bars indicate the first-order sensitivity indices, while the red bars represent total-effect sensitivity indices. The error bars show the bootstrap confidence intervals (95% confidence intervals) of the mean values.


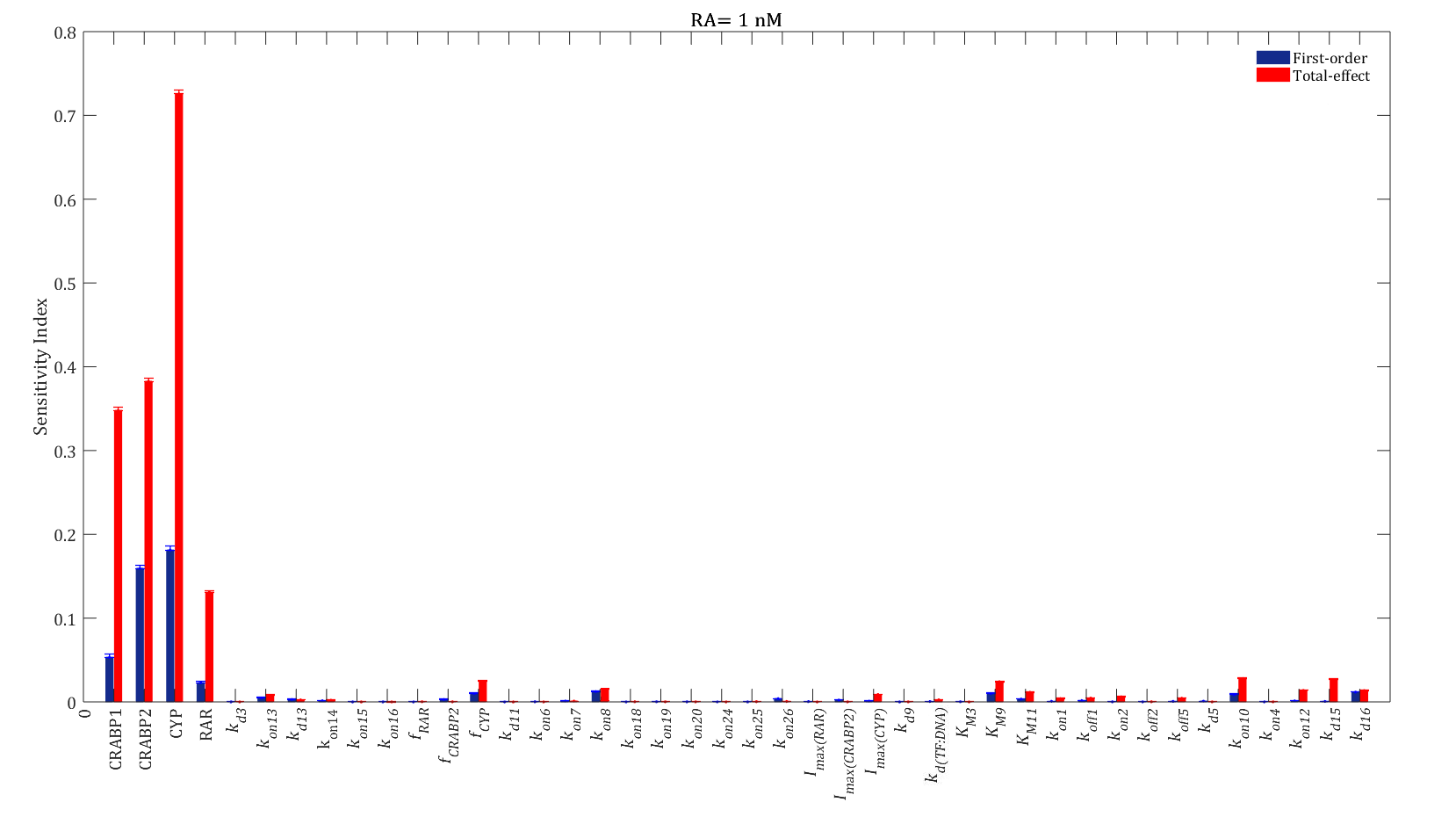


Figure S2. (a)


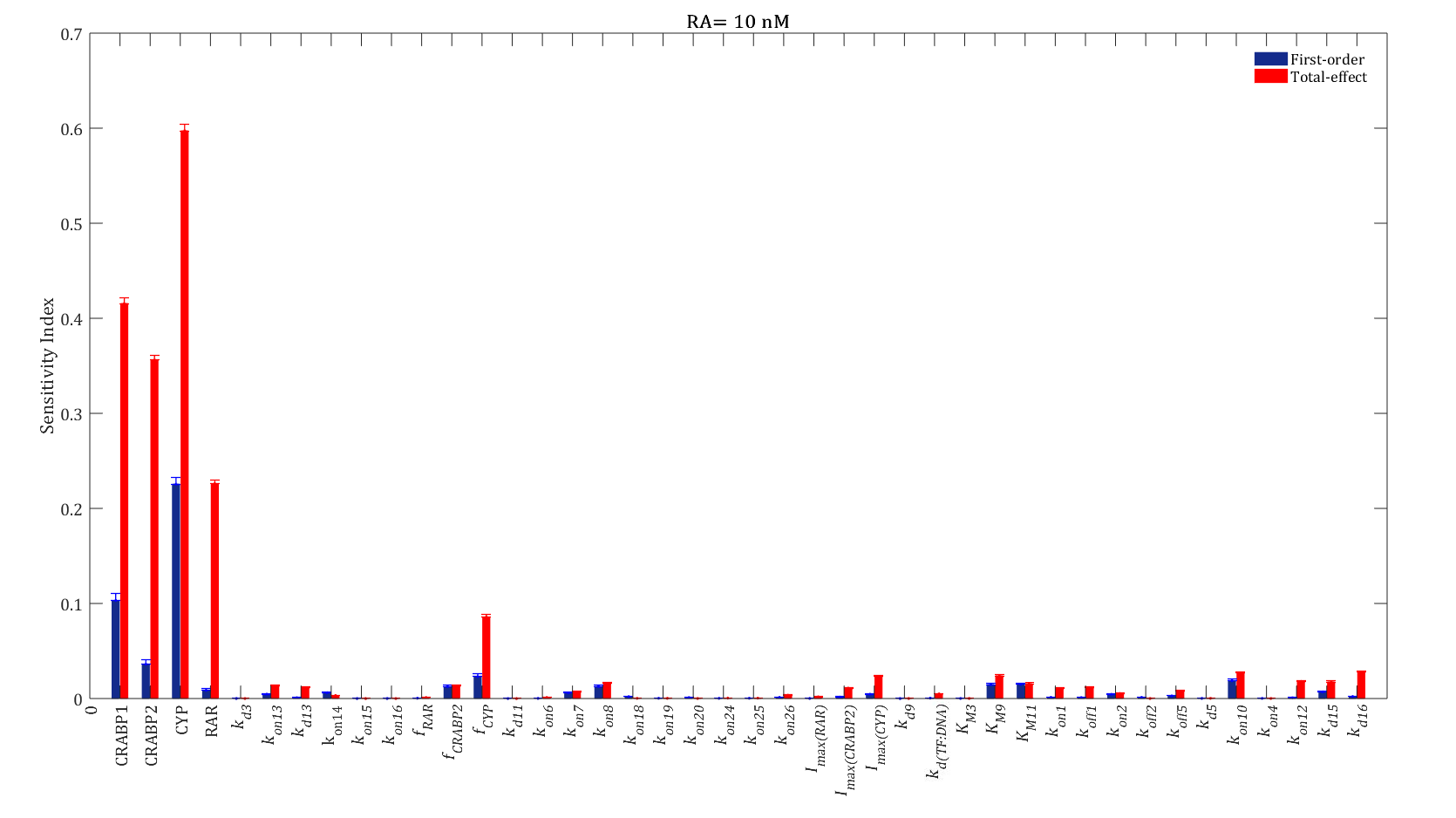


Figure S2. (b)


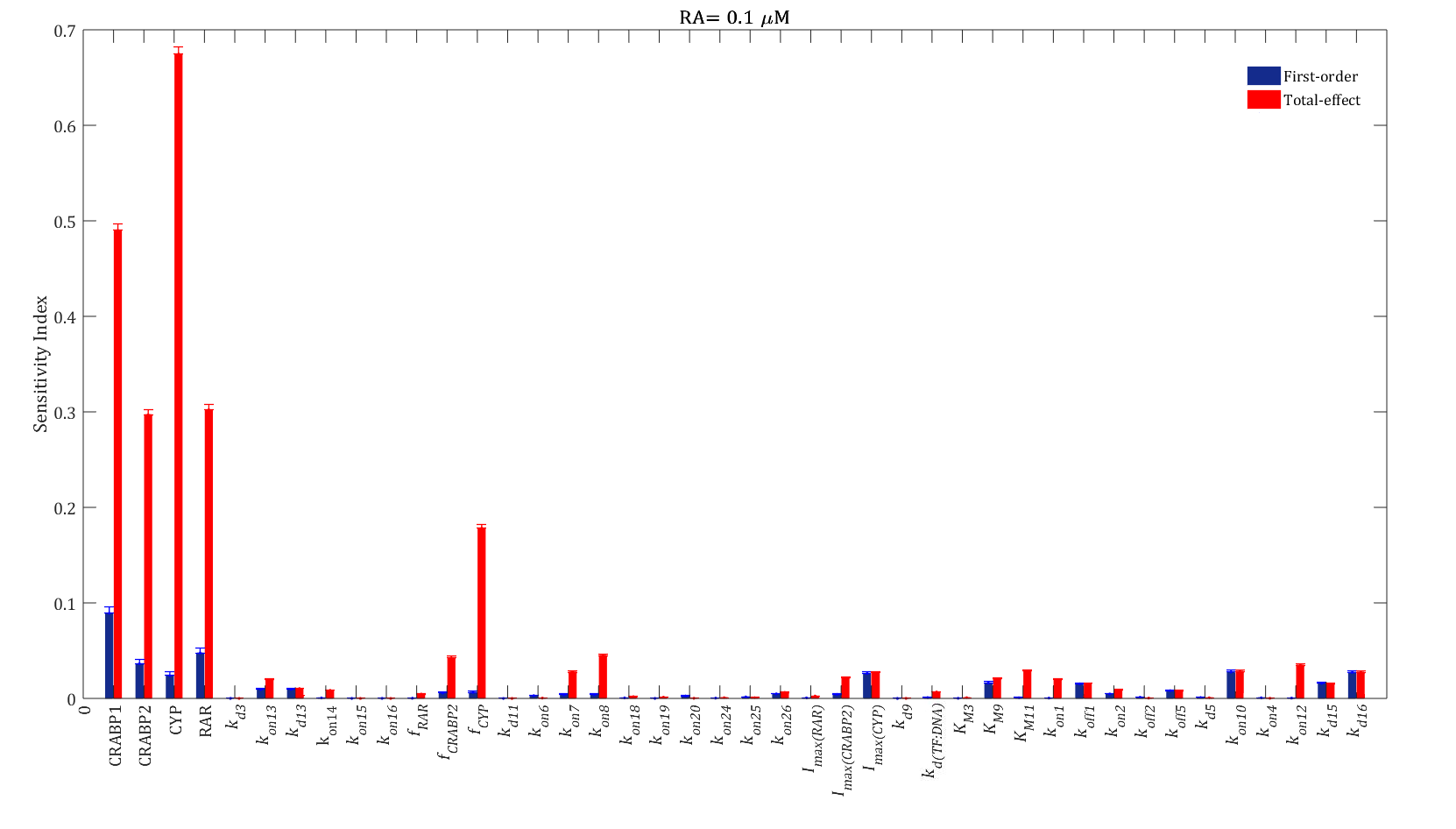


Figure S2. (c)


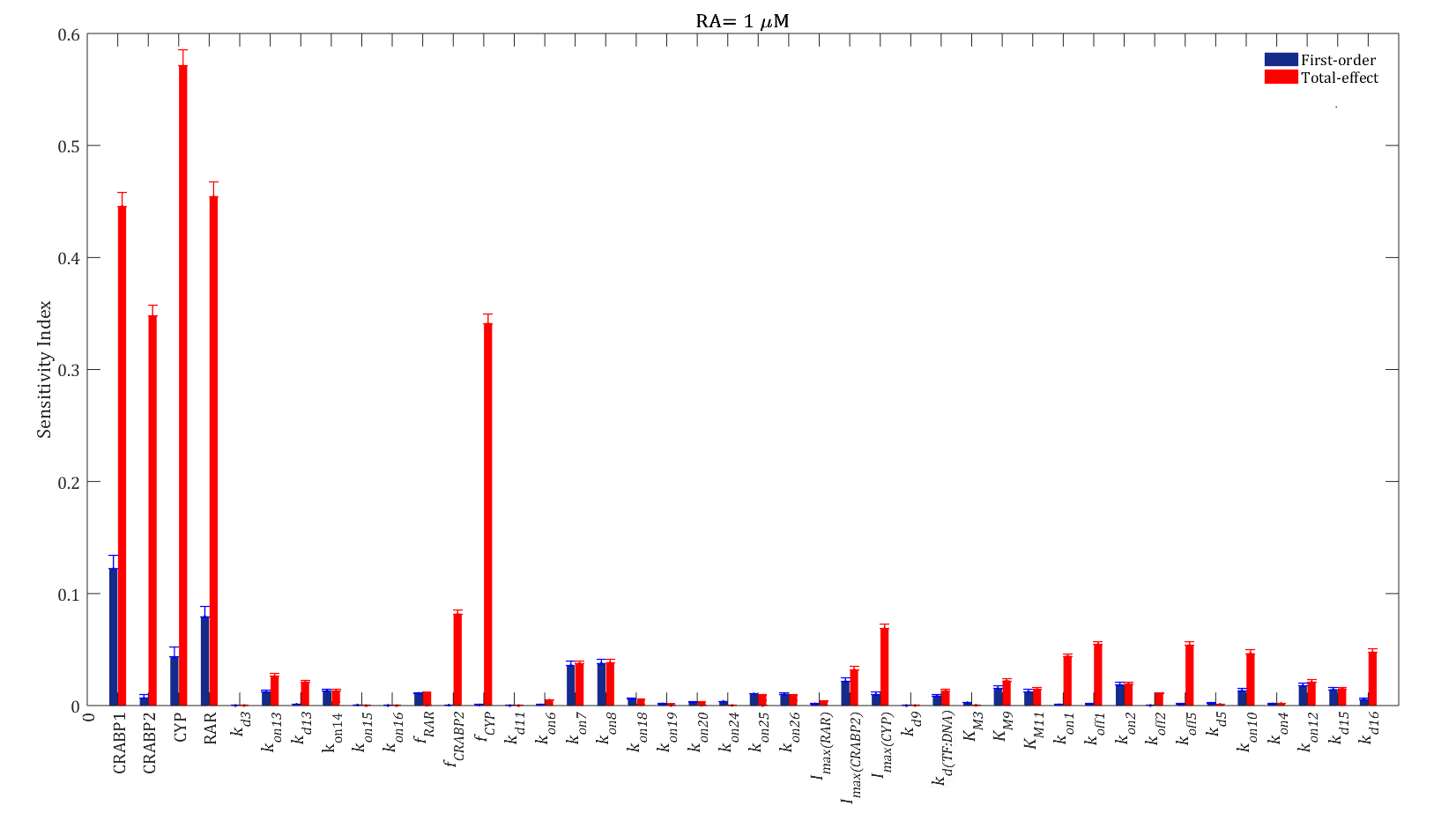


Figure S2. (d)

Figure S2. Sensitivity ranking of the model parameters. The model output was set to the time integral of the total RA metabolite formation within 24 hours after adding (a) 1 nM, (b) 10 nM, (c) 0.1 µM and (d) 1 µM of RA to the model. Blue bars indicate the first-order sensitivity indices, while the red bars represent total-effect sensitivity indices. The error bars show the bootstrap confidence intervals (95% confidence intervals) of the mean values.


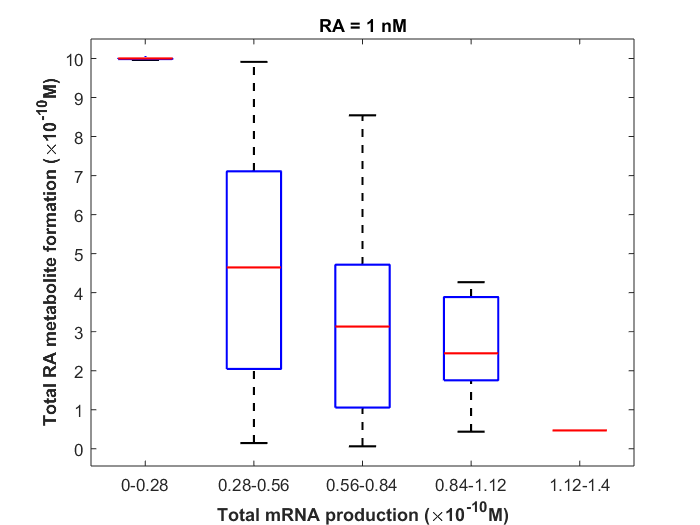


Figure S3. (a)


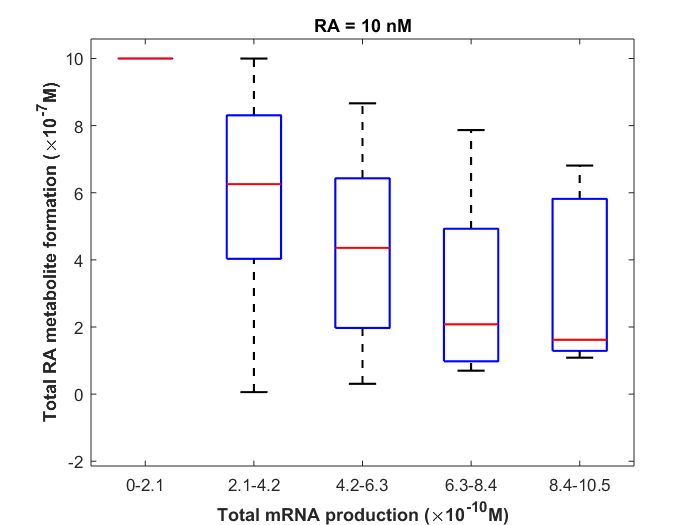


Figure S3. (b)


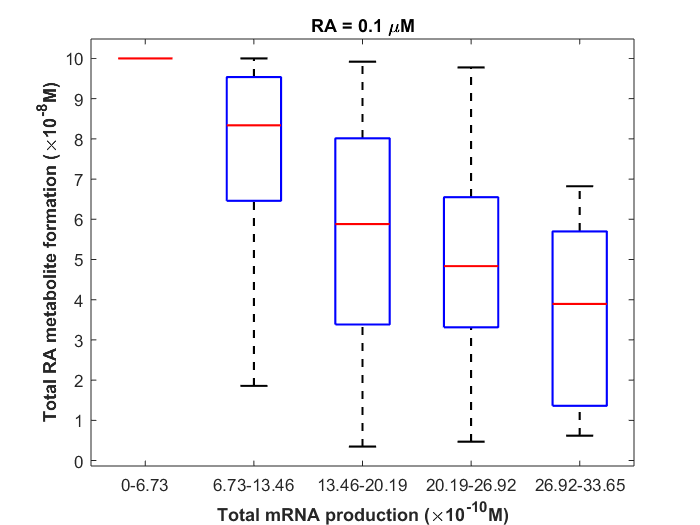


Figure S3. (c)

Figure S3. The relationship between total RA metabolite formation and total mRNA production within 24 hours of treatment with (a) 1 nM, (b) 10 nM, (c) 0.1 µM of RA. The results were obtained by random sampling of 10000 points following a uniform distribution over a 44-dimensional parameter space. The Spearman’s correlation coefficients and P values were (a) ρ=-0.44, p=0, (b) ρ=-0.5, p=0, (c) ρ=-0.63, p=0.

**References**

1. Nelson CH, Peng CC, Lutz JD, Yeung CK, Zelter A, Isoherranen N. Direct protein–protein interactions and substrate channeling between cellular retinoic acid binding proteins and CYP26B1. FEBS lett. 2016;590(16):2527-35.

2. Angulo A, Chandraratna RA, LeBlanc JF, Ghazal P. Ligand induction of retinoic acid receptors alters an acute infection by murine cytomegalovirus. J Virol. 1998;72(6):4589-600.

3. Ingalls BP. Mathematical modeling in systems biology: an introduction: MIT press; 2013.

4. Sauro HM. Enzyme kinetics for systems biology: Future Skill Software; 2011.

5. Stormo GD, Zhao Y. Determining the specificity of protein–DNA interactions. Nat Rev Genet. 2010;11(11):751-60.

6. Schwanhäusser B, Busse D, Li N, Dittmar G, Schuchhardt J, Wolf J, et al. Global quantification of mammalian gene expression control. Nat. 2011;473(7347):337-42.

7. Balmer JE, Blomhoff R. Gene expression regulation by retinoic acid. J Lipid Res. 2002;43(11):1773-808.

8. Fuchs G, Voichek Y, Benjamin S, Gilad S, Amit I, Oren M. 4sUDRB-seq: measuring genomewide transcriptional elongation rates and initiation frequencies within cells. Genome Biol. 2014;15(5):R69.

9. Zody MC, Garber M, Adams DJ, Sharpe T, Harrow J, Lupski JR, et al. DNA sequence of human chromosome 17 and analysis of rearrangement in the human lineage. Nat. 2006;440(7087):1045-9.

10. Gregory S, Barlow K, McLay K, Kaul R, Swarbreck D, Dunham A, et al. The DNA sequence and biological annotation of human chromosome 1. Nat. 2006;441(7091):315-21.

11. Hillier LW, Graves TA, Fulton RS, Fulton LA, Pepin KH, Minx P, et al. Generation and annotation of the DNA sequences of human chromosomes 2 and 4. Nat. 2005;434(7034):724-31.

12. Halford SE, Marko JF. How do site‐specific DNA‐binding proteins find their targets? Nucleic acids Res. 2004;32(10):3040-52.

13. Crettaz M, Baron A, Siegenthaler G, Hunziker W. Ligand specificities of recombinant retinoic acid receptors RAR α and RAR β. Biochem J. 1990;272(2):391-7.

14. Mirny L, Slutsky M, Wunderlich Z, Tafvizi A, Leith J, Kosmrlj A. How a protein searches for its site on DNA: the mechanism of facilitated diffusion. J Phys A Math Theor. 2009;42(43):434013.

15. Giguere V, Lyn S, Yip P, Siu C-H, Amin S. Molecular cloning of cDNA encoding a second cellular retinoic acid-binding protein. PNAS. 1990;87(16):6233-7.

16. Pianosi F, Sarrazin F, Wagener T. A Matlab toolbox for global sensitivity analysis. Environ Modell Software. 2015;70:80-5.

17. Zi Z. Sensitivity analysis approaches applied to systems biology models. IET Syst. Biol. 2011;5(6):336-46.

18. Zhang XY, Trame M, Lesko L, Schmidt S. Sobol sensitivity analysis: a tool to guide the development and evaluation of systems pharmacology models. CPT: pharmacometrics Syst Pharmacol. 2015;4(2):69-79.

19. Mokhtari A, Frey HC, Zheng J. Evaluation and recommendation of sensitivity analysis methods for application to stochastic human exposure and dose simulation models. J Exposure Sci Environ Epidemiol. 2006;16(6):491-506.

20. Tang T, Reed P, Wagener T, Van Werkhoven K. Comparing sensitivity analysis methods to advance lumped watershed model identification and evaluation. Hydrol Earth Syst Sci Discuss. 2006;3(6):3333-95.

21. Sin G, Gernaey KV, Neumann MB, van Loosdrecht MC, Gujer W. Global sensitivity analysis in wastewater treatment plant model applications: prioritizing sources of uncertainty. Water Res. 2011;45(2):639-51.

22. Cosenza A, Mannina G, Vanrolleghem PA, Neumann MB. Global sensitivity analysis in wastewater applications: A comprehensive comparison of different methods. Environ modell Software. 2013;49:40-52.

23. Dong D, Ruuska SE, Levinthal DJ, Noy N. Distinct roles for cellular retinoic acid-binding proteins I and II in regulating signaling by retinoic acid. J Biol Chem 1999;274(34):23695-8.

24. Kersten S, Dawson MI, Lewis BA, Noy N. Individual subunits of heterodimers comprised of retinoic acid and retinoid X receptors interact with their ligands independently. Biochem. 1996;35(12):3816-24.

25. Cambridge SB, Gnad F, Nguyen C, Bermejo JL, Krüger M, Mann M. Systems-wide proteomic analysis in mammalian cells reveals conserved, functional protein turnover. J proteome Res. 2011;10(12):5275-84.

26. Boskovic G, Desai D, Niles RM. Regulation of retinoic acid receptor α by protein kinase C in B16 mouse melanoma cells. J Biol Chem. 2002;277(29):26113-9.

27. Tsou HC, Lee X, Si SP, Peacocke M. Regulation of retinoic acid receptor expression in dermal fibroblasts. Exp Cell Res. 1994;211(1):74-81.

28. Redfern C, Lovat P, Malcolm A, Pearson A. Differential effects of 9-cis and all-trans retinoic acid on the induction of retinoic acid receptor-β and cellular retinoic acid-binding protein II in human neuroblastoma cells. Biochem J. 1994;304(1):147-54.
